# Transcriptional and metabolic effects of glucose on *Streptococcus pneumoniae* sugar metabolism

**DOI:** 10.3389/fmicb.2015.01041

**Published:** 2015-10-07

**Authors:** Laura Paixão, José Caldas, Tomas G. Kloosterman, Oscar P. Kuipers, Susana Vinga, Ana R. Neves

**Affiliations:** ^1^Laboratory of Lactic Acid Bacteria and In Vivo NMR, Instituto de Tecnologia Química e Biológica, Universidade Nova de LisboaOeiras, Portugal; ^2^Center of Intelligent Systems, Instituto de Engenharia Mecânica, Instituto Superior Técnico, Universidade de LisboaLisboa, Portugal; ^3^Department of Molecular Genetics, Groningen Biomolecular Sciences and Biotechnology Institute, University of GroningenGroningen, Netherlands

**Keywords:** *Streptococcus pneumoniae*, glycan-derived sugars, glucose, transcriptome, *in vivo*^13^C-NMR

## Abstract

*Streptococcus pneumoniae* is a strictly fermentative human pathogen that relies on carbohydrate metabolism to generate energy for growth. The nasopharynx colonized by the bacterium is poor in free sugars, but mucosa lining glycans can provide a source of sugar. In blood and inflamed tissues glucose is the prevailing sugar. As a result during progression from colonization to disease *S. pneumoniae* has to cope with a pronounced shift in carbohydrate nature and availability. Thus, we set out to assess the pneumococcal response to sugars found in glycans and the influence of glucose (Glc) on this response at the transcriptional, physiological, and metabolic levels. Galactose (Gal), N-acetylglucosamine (GlcNAc), and mannose (Man) affected the expression of 8 to 14% of the genes covering cellular functions including central carbon metabolism and virulence. The pattern of end-products as monitored by *in vivo* ^13^C-NMR is in good agreement with the fermentation profiles during growth, while the pools of phosphorylated metabolites are consistent with the type of fermentation observed (homolactic vs. mixed) and regulation at the metabolic level. Furthermore, the accumulation of α-Gal6P and Man6P indicate metabolic bottlenecks in the metabolism of Gal and Man, respectively. Glc added to cells actively metabolizing other sugar(s) was readily consumed and elicited a metabolic shift toward a homolactic profile. The transcriptional response to Glc was large (over 5% of the genome). In central carbon metabolism (most represented category), Glc exerted mostly negative regulation. The smallest response to Glc was observed on a sugar mix, suggesting that exposure to varied sugars improves the fitness of *S. pneumoniae*. The expression of virulence factors was negatively controlled by Glc in a sugar-dependent manner. Overall, our results shed new light on the link between carbohydrate metabolism, adaptation to host niches and virulence.

## Introduction

*Streptococcus pneumoniae* is a common asymptomatic commensal of the human nasopharynx, but also a life-threatening pathogen responsible for severe illnesses such as bacterial meningitis, pneumonia, septicaemia, as well as milder respiratory infections (Giammarinaro and Paton, 2002; Bogaert et al., 2004). The establishment of a carrier state (colonization) is a prerequisite for pneumococcal disease and an important feature for dissemination through the community (Bogaert et al., 2004; Kadioglu et al., 2008; King, 2010). Indeed, from the microbe's fitness perspective, the success of pneumococcal infections relies on colonization, multiplication and transmission to a new host (Hava et al., 2003). Consequently, the factors required for its commensal lifestyle might also be considered virulence factors (Hava et al., 2003; Weiser, 2010). The mechanisms underlying the progression from a carrier state to invasive disease are complex, probably multifactorial and are still poorly understood (Obaro and Adegbola, 2002; Ogunniyi et al., 2012). Notwithstanding, several studies based on different techniques to evaluate pneumococcal gene expression, revealed that pneumococcal virulence genes were differentially expressed in different host niches (Orihuela et al., 2000, 2004; Ogunniyi et al., 2002, 2009, 2012; LeMessurier, 2006; Mahdi et al., 2008). Therefore, we hypothesize that transition from carriage to disease involves modulation of pneumococcal gene expression in response to environmental changes. Indeed, it was shown that changes in the concentrations of metal ions in different host sites, contributes to virulence in *S. pneumoniae* (reviewed in Honsa et al., 2013; Shafeeq et al., 2013).

**Figure 1:**
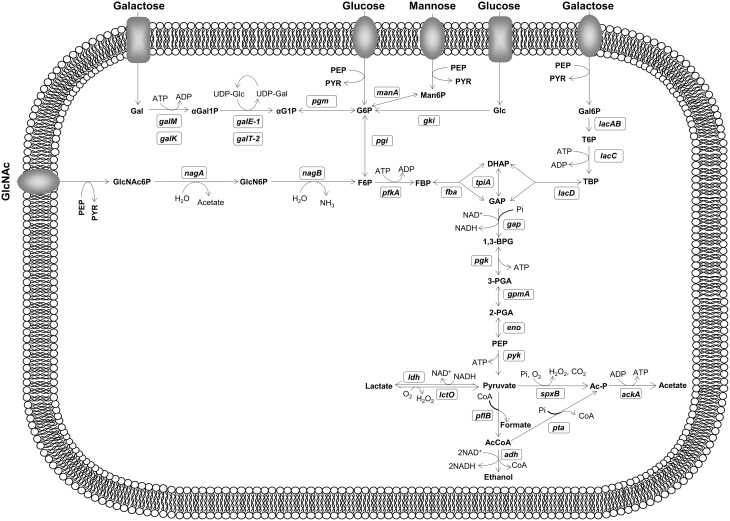
**Schematic representation of Gal, Man, GlcNAc, and Glc metabolism in ***S. pneumoniae*** D39**. Sugar-specific steps, glycolysis and fermentative metabolism are depicted. Putative and functional characterized genes encoding depicted metabolic steps are shown in white boxes. Proposed pathways were reconstructed based on genome information (http://www.ncbi.nlm.nih.gov/genomes/lproks.cgi), literature and database surveys (KEGG, MetaCyc). Gene annotation downloaded from NCBI: *gki*, glucokinase; *pgi*, glucose 6-phosphate isomerase; *pfkA*, 6-phosphofructokinase; *fba*, fructose bisphosphate aldolase; *tpiA*, triosephosphate isomerase; *gap*, glyceraldehyde-3-phosphate dehydrogenase; *pgk*, phosphoglycerate kinase; *gpmA*, phosphoglyceromutase; *eno*, phosphopyruvate hydratase (enolase); *pyk*, pyruvate kinase; *ldh*, L-lactate dehydrogenase; *lctO*, lactate oxidase; *spxB*, pyruvate oxidase; *ackA*, acetate kinase; *pta*, phosphotransacetylase; *pflB*, pyruvate formate-lyase; *adh* (spd_1834), bifunctional acetaldehyde-CoA/alcohol dehydrogenase; *galM*, aldose 1-epimerase; *galK*, galactokinase; *galE-1*, UDP-glucose 4-epimerase; *galT-2*, galactose 1-phosphate uridylyltransferase; *pgm*, phosphoglucomutase/phosphomannomutase family protein; *lacA*, galactose 6-phosphate isomerase subunit LacA; *lacB*, galactose 6-phosphate isomerase subunit LacB; *lacC*, tagatose 6-phosphate kinase; *lacD*, tagatose 1,6-diphosphate aldolase; *manA*, mannose 6-phosphate isomerase; *nagA*, N-acetylglucosamine 6-phosphate deacetylase; *nagB*, glucosamine 6-phosphate isomerase. Intermediates: G6P, glucose 6-phosphate; F6P, fructose 6-phosphate; FBP, fructose 1,6-biphosphate; GAP, glyceraldehyde 3-phosphate; DHAP, dihydroxyacetone phosphate; 1,3-BPG, 1,3-biphosphoglycerate; 3-PGA, 3-phosphoglycerate; 2-PGA, 2-phosphoglycerate; PEP, phospho*enol*pyruvate; GlcNAc6P, N-acetylglucosamine 6-phosphate; GlcN6P, glucosamine 6-phosphate; Man6P, mannose 6-phosphate; Gal, galactose; α-Gal, α-galactose; αGal1P, α-galactose 1-phosphate; αG1P, α-glucose 1-phosphate; UDP-Glc, UDP-glucose; UDP-Gal, UDP-galactose; Gal6P, galactose 6-phosphate; T6P, tagatose 6-phosphate; TBP, tagatose 1,6-diphosphate. Sugar transporters depicted as ovoids and rectangles generically represent phosphoenolpyruvate phosphotransferase systems or ABC transporters, respectively.

To a large extent pneumococcal pathogenesis relies on efficient acquisition and metabolism of carbohydrates required for growth and survival, but the knowledge of pneumococcal physiology and pathogenesis is still limited. *S. pneumoniae* is a strictly fermentative bacterium relying exclusively on carbohydrates to obtain energy for growth (Figure 1). Compared to other colonizers of its ecological niche like *Haemophilus influenzae* and *Neisseria meningitidis, S. pneumoniae* displays the broadest sugar utilization range (Tettelin et al., 2001). Analysis of the genome suggested the existence of pathways for catabolism of a wide diversity of carbohydrates (Tettelin et al., 2001) and a recent study showed that *S. pneumoniae* is able to use at least 32 substrates (Bidossi et al., 2012). In particular, the bacterium has catabolic pathways for the utilization of galactose (Gal), mannose (Man), and N-acetylglucosamine (GlcNAc) (Tettelin et al., 2001; Bidossi et al., 2012; Paixão et al., 2015). The pneumococcus possesses at least ten extracellular glycosidases, which enable the modification and breakdown of host glycans generating free sugars that can potentially be used for growth (reviewed by King, 2010). Furthermore, over 30% of all the transporters in the genome are presumably involved in sugar uptake (Tettelin et al., 2001; Bidossi et al., 2012), a by far larger proportion than that found in the other microorganisms occupying the same niche (Paulsen et al., 2000; Tettelin et al., 2001).

However, to thrive in diverse host environments, the bacterium has to cope with fluctuations in nature and availability of carbon sources. In the nasopharynx free sugars are scarce, but glycans are plentiful both in secretions and on the surface of epithelial cells. In contrast to the airways, the generally preferred sugar in *Streptococcaceae*, glucose (Glc), is present in comparatively higher concentrations in the bloodstream and during infection (Philips et al., 2003; Shelburne et al., 2008). We hypothesize that this metabolic flexibility is important during the transition from colonization to invasive disease in *S. pneumoniae*. To adapt to host environments, the pneumococcus has developed sophisticated and complex mechanisms. It has been proposed that exchanging genetic material (genetic transformation) provides a selective advantage in the adaptation of the pneumococcus to distinct environmental conditions (Claverys et al., 2000; Donati et al., 2010). Moreover, when exposed to a mixture of substrates bacteria can sense the nutritional environment and adjust its catabolic capabilities, in a process termed as carbon catabolite repression (CCR) (Titgemeyer and Hillen, 2002). CCR is a regulatory process that enables bacteria to utilize preferred carbon sources in detriment of others, by downregulating the expression of genes and inhibiting enzyme activities involved in the use of secondary carbon sources (Deutscher, 2008; Görke and Stülke, 2008). The molecular mechanisms mediating CCR are diverse (reviewed by Titgemeyer and Hillen, 2002; Deutscher, 2008; Görke and Stülke, 2008) and act at different regulatory layers: gene expression (transcription activation and gene repression), protein activities (control of translation, post-translational modification), and metabolites (allosteric regulation) (Görke and Stülke, 2008; Carvalho et al., 2011). In the Firmicutes, CCR typically comprises the general phospho*enol*pyruvate phosphotransferase system component HPr, the bifunctional HPr kinase/phosphorylase (HPrK), and the transcription factor catabolite control protein A (CcpA). The latter binds to catabolite response elements (CRE) in promoter regions of CCR-sensitive genes (Görke and Stülke, 2008).

Hence, a global understanding of the physiological response of *S. pneumoniae* to shifts in substrate availability requires the integration of diverse data sets collected at distinct “omic” levels. With the aim to study the response of *S. pneumoniae* to the monosaccharides present in host glycans we have collected transcriptome and time series metabolic data during growth on Gal, Man, and GlcNAc as single carbon sources and compared these to the profiles on Glc. Previously, we have shown that Glc supported the fastest growth followed by GlcNAc which was a better substrate than Gal or Man (Paixão et al., 2015). The markedly different growth profiles suggests that growth on these sugars is differently regulated. Furthermore, we investigated the effect of adding the fast metabolizable sugar (Glc) to *S. pneumoniae* cells actively growing on Gal, Man, GlcNAc, or in a mixture thereof, on the growth physiology and at the transcriptional and metabolic levels (by *in vivo* ^13^C-NMR, for Gal-adapted cells). Finally, we evaluated the impact of the substrate for growth on the expression of virulence factors.

In this work we have generated data at transcript-, metabolic-, and physiological levels, with regard to the response of *S. pneumoniae* to sugar availability. These data can in the future be used as input for predictive multi-level mathematical models of the pneumococcus metabolic networks. It is expected that such tools will facilitate our understanding of complex phenomena and enable the identification of novel targets for the development of therapeutics.

## Materials and methods

### Bacterial strains and growth conditions

*Streptococcus pneumoniae* strain D39 (serotype 2) and its derivative D39Δ*cps* (A. M. Cavaleiro, P. Gaspar, T. Kloosterman, O. P. Kuipers, and A. R. Neves., unpublished) were used throughout this study. The D39 isolate was obtained from the culture collection of the Department of Infection, Immunity and Inflammation, of the University of Leicester. Stocks were prepared as described elsewhere (Carvalho et al., 2013) and stored in glycerol (25% vol/vol) at −80°C. Working stocks were done by transferring 1 ml of the frozen stock to 25 ml Glc-M17 medium (Difco), followed by incubation at 37°C until late exponential phase (OD_600_ ~0.8). Cultures were centrifuged (6300 × *g*, 7 min, 4°C), the supernatant discarded and the pellet suspended in 20 ml 25% (vol/vol) glycerol-M17. 1 ml aliquots were stored at −80°C until further use.

Routinely, *S. pneumoniae* was grown statically in M17 broth (Difco) containing 0.5% (wt/vol) glucose (Glc-M17) at 37°C. For physiological studies and transcriptome analysis, *S. pneumoniae* D39 was grown in static rubber stoppered bottles at 37°C and without pH control (initial pH 6.5) in the chemically defined medium (CDM) described by Carvalho et al. (2013). Growth was performed as reported before (Paixão et al., 2015). Cells actively metabolizing single sugars Gal, GlcNAc, and Man (12–15 mM) or a sugar mixture (approximately 6 mM each, herein denominated sugar mix) were challenged or not with a 10 mM pulse of Glc at mid-exponential phase of growth. In the case of Glc challenge experiments, cells metabolizing other sugars were herein denominated “sugar”-adapted cells. Cultures were started by inoculating fresh CDM, to an initial optical density at 600 nm (OD_600_) of ~0.05, with a pre-culture grown until late exponential phase of growth. Pre-cultures were performed as described by Carvalho et al. (2013), except pre-cultures for growth on sugar mix, which were grown in CDM containing 30 mM of each carbon source. Growth was monitored by measuring OD_600_ hourly. Maximum specific growth rates (μ_max_) were calculated through linear regressions of the plots of ln(OD_600_) vs. time during the exponential phase of growth after the Glc pulse. The values reported are averages of two independent growth experiments.

### Quantification of substrate consumption and fermentation products

Strains were grown in CDM supplemented with the appropriate sugar as described above. Culture samples (2 mL) were taken at inoculation, immediately and 1 h after the Glc pulse, and at the onset of the stationary phase of growth, and centrifuged (16,100 × *g*, 3 min, 4°C). The supernatants were filtered (Q-Max® RR NY syringe 0.22 μm filters) and stored at −20°C until further analysis. Fermentation products, Glc and GlcNAc were quantified by high performance liquid chromatography (HPLC) equipped with a refractive index detector (Shodex RI-101, Showa Denko K. K., Japan) using an HPX-87H anion exchange column (Bio-Rad Laboratories Inc., California, USA) at 60°C, with 5 mM H_2_SO_4_ as the elution fluid and a flow rate of 0.5 ml min^−1^. Gal and Man were quantified by ^1^H-NMR and the spectra were acquired in a Bruker AMX300 spectrometer (Bruker BioSpin GmbH). To quantify Gal and Man the temperature of the probe was set to 18°C and to 37°C, respectively. DSS (3-(trimethylsilyl) propionic acid sodium salt) was added to the samples and used as an internal concentration standard in ^1^H-NMR quantifications.

Yields were calculated using the data from samples taken immediately after inoculation and at the onset of stationary phase of growth. A factor of 0.38, determined from a dry weight (DW) (mg ml^−1^) vs. OD_600_ curve, was used to convert OD_600_ into DW (mg biomass ml^−1^). The yield in biomass was calculated as g of dry weight per mol of substrate consumed. The ATP yield was determined as the ratio of ATP produced to substrate consumed at the time of growth arrest assuming that all ATP was synthesized by substrate-level phosphorylation. The values reported are averages of two independent growths.

### Transcriptome analysis

The transcript levels of *S. pneumoniae* D39 growing in CDM supplemented with Gal, Man, or GlcNAc were compared by transcriptome analysis to Glc-grown cells. Additionally, cells of D39 grown on a single sugar (Gal, Man, or GlcNAc) or in their mixture challenged with a Glc pulse (10 mM) were compared to unchallenged cells using whole-genome *S. pneumoniae* DNA microarrays (Kloosterman et al., 2006). Cells were harvested by centrifugation (7197 × *g*, 2.5 min, at room temperature) at exponential phase of growth 1 h after the pulse challenge that was given at mid-exponential growth. Cell pellets were suspended in the remaining medium, frozen in liquid nitrogen and stored at −80°C. mRNA isolation, synthesis, and labeling of cDNA and hybridization were performed as described before (Kloosterman et al., 2006). RNA extraction was performed from two independent cultures. Microarray experiments and analysis were done essentially as described elsewhere (van Hijum et al., 2005; Kloosterman et al., 2006). Samples were hybridized to microarray slides containing 3 spots per gene and covering the entire D39 genome.

In all cases, genes were considered significantly differentially expressed when the *p*-value was < 0.05/n. The Bonferroni correction factor *n* = 1769 × 7 corresponds to the total number of differential expression significance tests performed and thus accounts for multiple hypotheses testing. Overrepresentation of COG categories and MetaCyc pathways (with more than five genes) among significant genes was assessed via hypergeometric tests with a *p-value* threshold of *p* < 0.05/n, with the Bonferroni correction factor *n* = 63 × 7 corresponding to the total number of overrepresentation tests performed.

Heatmaps were generated by taking into account the genes that were differentially expressed in each sample subset (single sugars vs. Glc and Glc challenged experiments vs. unchallenged cells), and intersecting those genes with the annotated subset categories: sugar transporters and sugar catabolic genes (reviewed by Paixão et al., 2015), glycolytic genes and genes devoted to pyruvate metabolism (according to NCBI annotation) and virulence factors (Table S1).

Venn diagrams were generated with the Venny tool: http://bioinfogp.cnb.csic.es/tools/venny/. When creating the gene lists, we consider D39 genes that are differentially expressed in each conditions, regardless of the direction of differential expression. Microarray data have been deposited to the Gene Expression Omnibus (GEO) and have accession number GSE70648.

### *In vivo* ^13^C-NMR experiments with resting cells

Cells of *S. pneumoniae* D39Δ*cps* were grown under anaerobic conditions, in a 2-L bioreactor (Sartorius Biostat® B plus) in CDM supplemented with 55 mM of Gal or GlcNAc. On Man, the biomass generated was insufficient for NMR studies, hence the cells were grown in presence of 55 mM of Glc supplemented with 0.5 mM of Man. Growth was performed with controlled pH (6.5) and temperature (37°C), under anaerobic conditions essentially as described before (Carvalho et al., 2013). The medium was aseptically degassed with argon during 60 min before inoculation. Cultures were kept homogenized by using an agitation speed of 50 rpm. Cells were harvested (5750 × *g*, 7 min, 4°C) in the late-exponential phase of growth and suspensions prepared essentially as described elsewhere (Neves et al., 1999). In brief, cells were washed twice (5750 × *g*, 5 min, 4°C) with 50 mM KPi buffer (pH 6.5) supplemented with 1% (wt/vol) choline and suspended in 35 ml of the same buffer with 6% (vol/vol) of deuterium oxide.

*In vivo* ^13^C-NMR experiments were performed online under controlled conditions of pH (6.5), temperature (37°C) and atmosphere (anaerobic conditions, argon atmosphere), using the circulating system as described by Neves et al. (1999). Substrates specifically labeled with ^13^C at carbon one (20 mM) were added to the cell suspension at time zero and spectra (30 s) acquired sequentially. For the two pulse substrate experiment, cells of D39Δ*cps* actively metabolizing 20 mM of [1-^13^C]Gal were challenged with a 10 mM pulse of [2-^13^C]Glc. ^13^C enrichment in different carbons allows traceability of the substrates in deriving intracellular metabolites and end-products. After substrate depletion and when no changes in the resonances due to end-products and intracellular metabolites were observed, the NMR experiment was stopped and a total cell extract was prepared by passing the cell suspension three times through the French press (6.21 MPa); the resulting total extract was incubated 15 min at 80–90°C and cooled down on ice. Cell debris and denaturated macromolecules were removed by centrifugation (45696 × *g*, 10 min, 4°C), and the supernatant (herein designated as NMR cell extract) was used for metabolite quantification of end-products and minor metabolites that remained inside the cells, which was accomplished in fully relaxed ^13^C-spectra, at 30°C. The lactic acid and acetate produced were quantified in the NMR cell extract by ^1^H-NMR on a Bruker AMX300 spectrometer (Bruker Biospin GmbH), using formic acid (sodium salt) as an internal concentration standard (Neves et al., 1999).

Due to fast pulsing conditions during *in vivo* ^13^C-spectra acquisition, correction factors were determined allowing the conversion of peak intensities into concentration of intracellular metabolites. Correction factors for resonances due to C1 and C6 of FBP were determined (0.73 ± 0.07) to convert peak intensities to concentrations as described by Neves et al. (2002a), but the temperature was kept at 37°C. Resonances due to C1 α-mannose 6-phosphate (α-Man6P) and C1 β-mannose 6-phosphate (β-Man6P) were determined (0.33 and 0.51, respectively), at 37°C. A value of 3 μl (mg protein)^−1^ of intracellular volume of *S. pneumoniae* was used to determine the intracellular concentrations of metabolites (Ramos-Montañez et al., 2010). The concentration limit for detection of intracellular metabolites under the conditions employed was 3–4 mM. For dry mass determination, 1 ml of cell suspension obtained after *in vivo* ^13^C NMR experiment was filtered through 0.22 μm pore size membranes, dried at 100°C and desiccated for 45 min prior weighing. This was done in duplicates for each experiment. The quantitative kinetic data for intracellular metabolites were determined as described elsewhere (Neves et al., 1999). The values presented are averages of at least two independent assays.

### Identification of transient resonances

Transient resonances observed during *in vivo* NMR experiments were assigned by spiking pure compound to NMR extracts obtained from actively metabolizing cell suspensions. In brief, during the metabolism of the labeled substrate, 1 ml aliquot was withdrawn and perchloric acid (0.6 M, final concentration) was added. After 20 min stirring on ice the pH was set to neutral with 2M KOH, and the cell extract was centrifuged (60 min, 4°C, 16100 × *g*). The supernatant was frozen with liquid nitrogen, lyophilized, and suspended in bi-distilled water.

### NMR spectroscopy

^13^C spectra were acquired at 125.77 MHz using a quadruple nuclei probe head on a Bruker AVANCE II 500 MH spectrometer (Bruker Biospin GmbH, Karlsruhe, Germany), as described before (Neves et al., 1999).

Determination of the correction factors was accomplished by acquisition of ^13^C-NMR spectra with a 60° flip angle and a recycle delay of 1.5 s, for saturating conditions or 60.5 s (relaxed conditions). For assignment of unknown compounds, carbon NMR spectra were recorded using a selective de carbon probe head (^13^C-Dual).

Carbon chemical shifts are referenced to the resonances of external methanol, designated at 49.3 ppm.

### Chemicals

Galactose and mannose were purchased from Sigma-Aldrich. Glucose was supplied by Merck and N-acetylglucosamine was purchased from Applichem. [1-^13^C] labeled compounds (galactose, glucose, mannose, and N–acetylglucosamine) and [2-^13^C]glucose, 99% isotopic enrichment, were obtained from Cortecnet. DSS and formic acid (sodium salt) were purchased from Merck. All other chemicals used were reagent grade.

## Results and discussion

### Transcriptional and metabolic changes during growth on glycan-derived sugars

We have previously shown the aptitude of *S. pneumoniae* D39 to use GlcNAc, Gal and Man as sole carbon sources for growth (Paixão et al., 2015). The growth profiles sustained by these sugars are markedly different, which already indicates differential gene expression. Thus, to investigate the effect of the glycan-derived monosaccharides on gene expression, a whole-genome transcriptome analysis was conducted, in which the transcript levels of cells grown on Gal, Man or GlcNAc were compared to those of Glc-grown cells (Tables S2–S4). The results are summarized in Table 1. A detailed description of the transcriptome results is given in Supplementary Text 1, Figure S1.

**Table 1:** **Significantly differentially expressed genes (up- or downregulated) in cells of ***S. pneumoniae*** D39 grown in CDM supplemented with N-acetylglucosamine (GlcNAc), mannose (Man), or galactose (Gal) as compared to glucose (Glc), determined by DNA microarrays^a^**.

**Category**	**Locus_tag**	**Gene**	**Product**	**Up- or down- regulation^b^**		
				**GlcNAc**	**Man**	**Gal**
**SUGAR-SPECIFIC CATABOLISM^c^**						
	**SPD_0071**	***galM***	**Aldose 1-epimerase**			**0.58**
	**SPD_1050**	***lacD***	**Tagatose 1,6-diphosphate aldolase**			**3.00**
	**SPD_1051**	***lacC***	**Tagatose-6-phosphate kinase**			**2.87**
	**SPD_1052**	***lacB***	**Galactose-6-phosphate isomerase subunit LacB**			**3.05**
	SPD_1163		N-acetylneuraminate lyase, putative	1.39	0.75	
	**SPD_1246**	***nagB***	**Glucosamine-6-phosphate isomerase**		0.46	
	**SPD_1432**	***galE-1***	**UDP-glucose 4-epimerase**			**0.77**
	SPD_1488		ROK family protein	1.58	0.83	
	**SPD_1612**	***galE-2***	**UDP-glucose 4-epimerase**			**−0.57**
	**SPD_1633**	**galT-2**	**Galactose-1-phosphate uridylyltransferase**			**3.11**
	**SPD_1634**	***galK***	**Galactokinase**		0.64	**3.30**
	**SPD_1866**	***nagA***	**N-acetylglucosamine-6-phosphate deacetylase**			0.47
	SPD_1993	*fucU*	RbsD/FucU transport protein family protein		−0.98	
	SPD_1994	*fucA*	L-fuculose phosphate aldolase		−0.82	
	SPD_1995	*fucK*	L-fuculose kinase FucK, putative		−1.25	
**GLYCOLYSIS**						
	SPD_0445	*pgk*	Phosphoglycerate kinase		0.46	
	SPD_0526	*fba*	Fructose-bisphosphate aldolase		0.45	
	SPD_0790	*pyk*	Pyruvate kinase		0.57	
	SPD_1012	*eno*	Enolase		0.61	
	SPD_1823	*gap*	Glyceraldehyde-3-phosphate dehydrogenase		0.53	
**DOWNSTREAM PYRUVATE**						
	SPD_0420	*pflB*	Pyruvate formate-lyase		1.25	
	SPD_0621	*lcto*	Lactate oxidase		0.38	
	SPD_0985	*pta*	Phosphotransacetylase			0.64
	SPD_1834	*adh*	Bifunctional acetaldehyde-CoA/alcohol dehydrogenase		1.00	
**SUGAR-SPECIFIC TRANSPORTERS^c^**						
	**SPD_0088**		**ABC transporter, permease protein**			**−0.73**
	**SPD_0089**		**ABC transporter, permease protein**			**−0.80**
	SPD_0279	*celB*	Cellobiose phosphotransferase system IIB component	−2.13	−1.80	−2.41
	SPD_0281	*celC*	Cellobiose phosphotransferase system IIA component	−2.30	−1.98	−2.29
	SPD_0283	*celD*	Cellobiose phosphotransferase system IIC component	−1.91	−2.24	−1.61
	SPD_0360	*mtlA*	PTS system, mannitol-specific IIBC components			0.62
	SPD_0502		PTS system, beta-glucosides-specific IIABC components	−0.65	−0.47	−0.73
	**SPD_0559**		**PTS system IIA component, putative**			**1.26**
	**SPD_0560**		**PTS system, IIB component, putative**			**1.21**
	**SPD_0561**		**PTS system, IIC component, putative**			**1.55**
	**SPD_0661**	***exp5***	**PTS system, IIABC components**	**−0.61**		
	SPD_0773		PTS system, fructose specific IIABC components		0.36	
	SPD_1039	*ptsI*	Phosphoenolpyruvate-protein phosphotransferase		0.49	
	SPD_1040	*ptsH*	Phosphocarrier protein HPr		0.56	
	SPD_1047	*lacE-2*	PTS system, lactose-specific IIBC components			0.78
	SPD_1057		PTS system, IIB component, putative			1.48
	SPD_1409		Sugar ABC transporter, ATP-binding protein			−0.52
	SPD_1493		Sugar ABC transporter, permease protein		0.61	
	SPD_1494		Sugar ABC transporter, permease protein		0.54	
	SPD_1495		Sugar ABC transporter, sugar-binding protein		0.56	
	SPD_1496		PTS system, IIBC components		−0.48	−0.72
	SPD_1832		PTS system, IIB component		−0.54	
	SPD_1833		PTS system, IIA component		−0.57	
	SPD_1959	*ulaA*	Ascorbate-specific PTS system enzyme IIC			−0.51
	**SPD_1989**		**PTS system, IID component**		**−0.63**	
	**SPD_1991**		**PTS system, IIB component**		**−0.78**	
	**SPD_1992**		**PTS system, IIA component**		**−0.76**	

a *Subtable of Tables S2–S4*.

b *Values of ln-ratio. Positive values indicate upregulation and negative values indicate downregulation*.

c *As reviewed by Paixão et al. (2015)*.

*In bold are depicted the transporters and sugar-specific catabolic genes known or putatively involved in the metabolism of GlcNAc, Man, or Gal, as reviewed by Paixão et al. (2015)*.

**Figure 2:**
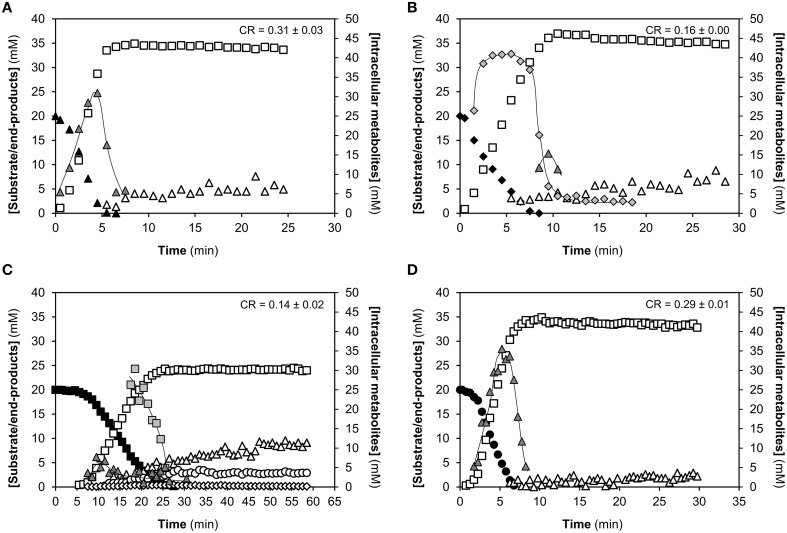
**Kinetics of 20 mM [1-^13^C]-sugar consumption, end-products formation and metabolic intermediates derived from the catabolism of different carbon sources by non-growing cells of ***S. pneumoniae*** D39Δ***cps*****. **(A)** N-acetylglucosamine (GlcNAc), **(B)** mannose (Man), **(C)** galactose (Gal), and **(D)** glucose (Glc). The metabolism was monitored online by *in vivo* ^13^C-NMR. Experiments were carried out at 37°C, under anaerobic conditions and pH control (6.5). Figures are from representative experiments from at least 2 replicates. The pyruvate concentration is depicted as extracellular concentration. Lines associated to intracellular metabolite time courses are simple interpolations. CR, maximal substrate consumption rate; FBP, fructose 1,6-biphosphate; Man6P, mannose 6-posphate; α-Gal6P, α-galactose 6-phosphate. Symbols: (▴), GlcNAc; (♦), Man; (■), Gal; (●), Glc; (**□**), lactate; (▵) acetate; (○), ethanol; (♢), pyruvate; (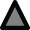), FBP; (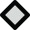), Man6P; (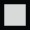), α-Gal6P.

Moreover, the remarkably different growth phenotypes might reflect the existence of metabolic constraints in sugar catabolism. Hence, catabolism of GlcNAc, Man and Gal was investigated by *in vivo* ^13^C-NMR in non-growing suspensions of cells grown on the specific sugar under study. As described, a non-encapsulated derivative of strain D39 was used, D39Δ*cps*. The kinetics of sugar consumption and end-products formation during the catabolism of [1-^13^C]GlcNAc, Man and Gal are shown on Figure 2. Glucose catabolism is shown for comparison on Figure 2D.

#### N-acetylglucosamine

The fermentation profile in non-growing suspensions is homolactic as previously shown for growing cells (Paixão et al., 2015). Lactate (33.6 ± 1.1 mM), the main end-product from the fermentation of the amino sugar (20 mM), accounted for 84% of the substrate consumed. Acetate was also produced (4.0 ± 0.9 mM) as a catabolic product of GlcNAc. Pyruvate was detected *in vivo*, but in quantities that did not allow its reliable quantification. The maximal substrate consumption rate was 0.31 ± 0.03 μmol min^−1^ mg^−1^ of protein (Figure 2A), a value higher than on Gal or Man, but similar to that on Glc (Figure 2). In fermentative lactic acid bacteria, homolactic metabolism has generally been associated with fast metabolizable sugars (Garrigues et al., 1997). GlcNAc fermentation in *S. pneumoniae* seems to be no exception, but the molecular mechanisms underlying this behavior have yet to be disclosed (Figure 2).

**Figure 3:**
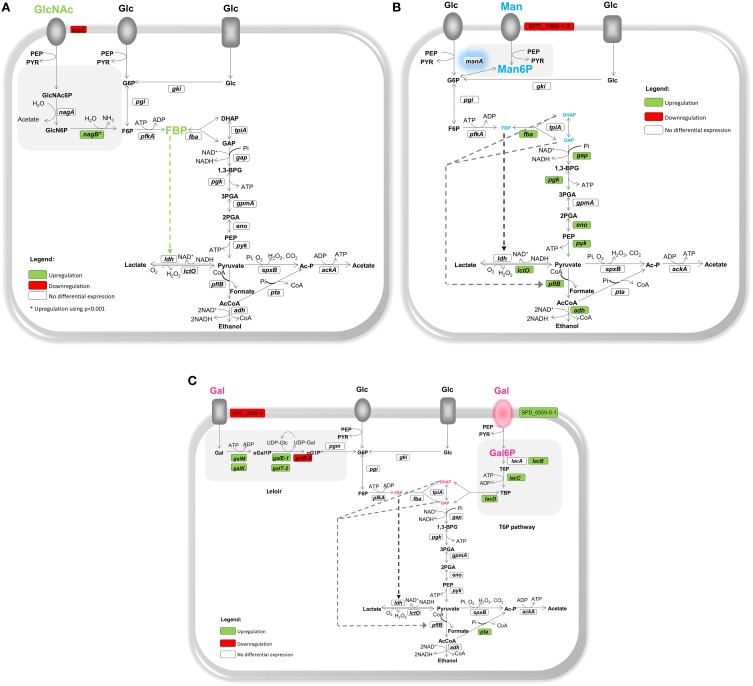
**Schematic representation of the model illustrating the physiological response of ***S. pneumoniae*** grown on glycan-derived sugars**. Transcriptional and metabolic changes in presence of **(A)** N-acetylglucosamine (GlcNAc), **(B)** mannose (Man), and **(C)** galactose (Gal) as compared to glucose (Glc) are shown. Target genes were those of central metabolism, glycolysis, and fermentation (pyruvate metabolism), and those encoding specific enzymes of glycan-derived catabolic pathways (transporters and catabolism). Gene annotations downloaded from NCBI, and intermediates are as in Figure 1. Sugar transporters depicted as ovoids and rectangles generically represent phosphoenolpyruvate phosphotransferase systems or ABC transporters, respectively. Green, red, and white boxes indicate upregulation, downregulation and no differential gene expression in glycan-derived grown cells as compared to Glc. ^*^ Upregulation using a less restrictive microarrays criterion (*p* < 0.001). Highlighted in green, blue, and pink are the glycolytic and/or sugar-specific catabolic intermediates detected by *in vivo* ^13^C-NMR in presence of GlcNAc, Man and Gal, respectively. The size of the font is indicative of the pool size of the intermediates (glycolytic or sugar-specific) accumulated in resting cells as determined by *in vivo* ^13^C-NMR. Big bold font indicates high accumulation whereas small size font is indicative of low amounts. DHAP and GAP are not detected by *in vivo* ^13^C-NMR but low accumulation in Man and Gal-grown cells, as compared to Glc-grown cells, is inferred due to the reversibility of the reactions catalyzed by FBP aldolase and triose 3-phosphate isomerase. Green dashed lines indicate positive metabolic regulation of enzymes. Dark gray dashed lines represent relief of LDH activation and light gray dashed lines represent relief of PFL inhibition. Blue shadow highlights a possible metabolic bottleneck in Man catabolism. Pink transporter highlights a possible bottleneck in Gal catabolism.

The glycolytic intermediate fructose 1,6-biphosphate (FBP) was the only intracellular metabolite detected in non-growing cells by *in vivo* NMR (Figure 2A). The kinetic profile resembles closely the accumulation of FBP during Glc metabolism (Figures 2A,D, Table 2), suggesting that regulation of glycolysis is similar for both sugars. In agreement, genes encoding activities in central carbon pathways, glycolysis, and fermentation (pyruvate conversion), were not significantly differentially expressed when comparing GlcNAc to Glc (Table 1 and Table S2, Figure 3A). Indeed, it is well documented that FBP accumulates to higher amounts during the catabolism of fast metabolizable sugars as Glc than less preferred carbohydrates. The reasons for the accumulation of this metabolite are diverse (Garrigues et al., 1997; Neves et al., 1999, 2002b; Ramos et al., 2004) and still matter of debate (reviewed by Neves et al., 2005).

**Table 2:** **Maximal concentrations of glycolytic and sugar-specific intermediates during the metabolism of N-acetylglucosamine (GlcNAc), mannose (Man), galactose (Gal), and glucose (Glc), by non-growing cells of ***S. pneumoniae*** D39Δ***Cps***, determined by ***in vivo***^13^C NMR**.

**Sugar**	**GlcNAc**	**Man**	**Gal**	**Glc^a^**	**Gal pulse Glc**
FBP_max_ (mM)	28.6 ± 2.2	11.7 ± 5.1	12.2 ± 6.4	35.0 ± 2.0	10.1 ± 4.1^b^/3.8 ± 0.5^c^ 8.0 ± 1.2^d^
Man6P_max_ (mM)	ND	37.1 ± 5.4	ND	ND	ND
α-Gal6P_max_ (mM)	ND	ND	36.1 ± 8.1	ND	39.0 ± 0.4/19.1 ± 3.4^e^

*Values determined by ^13^C-NMR in the NMR cell extracts from the cell suspensions used in the in vivo NMR*.

*ND, not detected in vivo or in the NMR cell extract. The values are averages of at least two independent experiments*.

a *Values reported by (A. M. Cavaleiro, P. Gaspar, T. G. Kloosterman, O. P. Kuipers and A. R. Neves., unpublished data)*.

b *Maximal FBP accumulation, derived from Gal metabolism*.

c *Maximal FBP accumulation, derived from Glc metabolism*.

d *Second FBP accumulation, derived from Gal metabolism*.

e *Second α-Gal6P accumulation, derived from Gal metabolism*.

*FBP, fructose 1,6-biphosphate; Man6P, mannose 6-phosphate; α-Gal6P, α-galactose 6-phosphate*.

Consumption of GlcNAc and Glc was identical in resting cells (Figure 2), but Glc supported much faster growth than GlcNAc (Paixão et al., 2015). The poor performance of GlcNAc in supporting growth combined with larger accumulation of phosphorylated metabolites in growing cells (Paixão et al., 2015 and Table S5) indicate a metabolic bottleneck in anabolic processes, whereas catabolic processes are identical for GlcNAc and Glc as evidenced from the *in vivo* ^13^C-NMR data. In agreement, glycolytic genes were not differentially expressed when comparing GlcNAc to Glc (Table S2, Figure 3A). The expression data supports this view and the homolactic profile in growing cells, but it fails to explain the lower growth rates on the amino sugar (about 1.7 times lower than on Glc) (Paixão et al., 2015). Growth rate is by far a more complex property, which cannot be fully explained by differences in the glycolytic flux that might (or not) arise from differentially expression of genes. A limiting step, however, can be the uptake of GlcNAc as induction of specific transporters was not observed. Indeed, neither GlcNAc specific transporters nor the dedicated catabolic genes (*nagA* and *nagB*) were altered, except for gene *exp5*, a putative GlcNAc PTS transporter, which is downregulated (Table 1 and Table S2, Figure 3A). In other bacteria, such as *Streptococcus mutans* or the model organisms *Bacillus subtilis* and *Escherichia coli*, GlcNAc induced the expression of genes encoding the specific catabolic pathway (Moye et al., 2014a). In a previous study, we showed that the activity of glucosamine 6-phosphate isomerase (NagB) was 4 times higher than that of N-acetylglucosamine 6-phosphate deacetylase (NagA) in GlcNAc-grown cells (Paixão et al., 2015). Re-evaluation of the microarray data using a less restrictive criterion (*p* < 0.001) showed that *nagB* was slightly induced by GlcNAc (1.40 times), but the expression of *nagA* was not altered. In *S. mutans*, the levels of *nagB* and *nagA* transcripts also differ (mRNA *nagB* > *nagA*) (Moye et al., 2014a), and this profile was interpreted as a mechanism to ensure a response to sugar variations while keeping the pools of GlcN6P to optimize growth. GlcN6P was identified as an allosteric effector that alleviates the repression of *nagA* and *nagB* mediated by the transcriptional regulator NagR.

#### Mannose

In Man metabolizing cells, lactate was the major end-product (30.4 ± 6.1 mM), accounting for 76% of the Man consumed. The acetate produced (9.0 ± 3.5 mM) was 4-fold higher than in Glc. Pyruvate was detected *in viv*o, but the low amounts hampered reliable quantification. The deviation toward mixed acid fermentation is consistent with the profile observed in Man-grown cells (Paixão et al., 2015), and substantiated by the upregulation of the fermentative genes encoding a bifunctional acetaldehyde-coA/alcohol dehydrogenase (*adh*) and pyruvate-formate lyase (*pflB*) (Table 1). However, a more pronounced shift could be expected, as fermentation of non-preferential sugars is generally associated with mixed acid profiles (Garrigues et al., 1997). Earlier, we reported Man as a non-preferential sugar for growth and that it supported the lowest growth rates under substrate excess (Paixão et al., 2015), and now we show that catabolism of Man (consumption rate 0.16 ± 0.00 μmol min^−1^ mg^−1^ of protein) is 2-times slower than that of Glc. What renders Man such a poor substrate is not clear, but the upregulation of glycolytic genes (Table 1), during growth on Man can be surmised as a cellular response to overcome Man-associated metabolic limitations (Table 1 and Table S3, Figure 3B).

Indeed, FBP and mannose 6-phosphate (Man6P) accumulated during the catabolism of Man (Figure 2B). The Man6P pool increased sharply to a steady concentration, which swiftly dropped to concentrations below 5 mM at the onset of Man depletion (Table 2, Figure 2B). In contrast, FBP became detectable after Man depletion and when the pool of Man6P was decreasing (Table 2, Figure 2B). Man6P is also the predominant phosphorylated metabolite in Man-grown cells (Table S5, Paixão et al., 2015). Induction of the glycolytic genes could thereby be a cellular response to alleviate the burden associated with the accumulation of the phosphorylated intermediate, allowing a more rapid flow through the central metabolism. Indeed, toxicity ascribed to sugar-phosphate accumulation has often been associated with defects or arrest of growth (Andersen et al., 2001; Vanderpool and Gottesman, 2007) and accumulation to high-levels of non-glycolytic phosphorylated metabolites is a recurrent observation during the metabolism of less preferred substrates (Neves et al., 2002c, 2006). Thus, it is tempting to suggest that Man6P toxicity results in lower glycolytic and growth rates. In line, a strain displaying higher Man6P isomerase (ManA) activity grew faster (1.6-fold) on Man than strain D39 (Paixão et al., 2015). For these reasons we proposed ManA as a metabolic bottleneck in strain D39 (Paixão et al., 2015). In fungi (*Saccharomyces cerevisiae* and *Aspergillus fumigatus*) deletion of the phosphomannose isomerase gene led to accumulation of Man6P, which decreased the glycolytic flux (Pitkänen et al., 2004; Fang et al., 2009). In *Corynebacterium glutamicum*, overexpression of *manA* alleviated the accumulation of Man6P (and F6P) and improved Man catabolism (Sasaki et al., 2011).

Alternatively, we can speculate that Man6P might exert repression over Man transporters, thus slowing down the uptake of this sugar and subsequent metabolism.

The general components of the PTS systems, phosphocarrier protein HPr (*ptsH*), and phosphoenolpyruvate-protein phosphotransferase, Enzyme I (*ptsI*), were induced during growth on Man (Table 1), indicating that Man translocation is primarily mediated by a PTS system. In agreement, a *ptsI* mutant of strain D39 showed practically no growth on Man, while inactivation of the PTS-Man (*manLMN*) dramatically reduced the ability of strain D39 to grow on mannose (A. M. Cavaleiro, P. Gaspar, T. G. Kloosterman, O. P. Kuipers, and A. R. Neves, unpublished data). In strain DP1004, a rough derivative of D39, mutation of *ptsI* partially reduced the growth on Man, but non-PTS systems for Man uptake were not ruled out (Bidossi et al., 2012).

The dedicated Man catabolic gene mannose 6-phosphate isomerase (*manA*) was not differentially expressed (even using a less restrictive criterion). The absence of significantly differentially expressed sugar-specific catabolic genes in the presence of Man and GlcNAc might reflect the constitutive expression of these genes, as their activities provide precursors for biosynthesis. In accordance, we have shown activity of ManA and NagA in Glc-grown cells (Paixão et al., 2015). However, we have also reported that in presence of the inducing sugar their activities were considerably higher (Paixão et al., 2015). The lack of correlation between the transcript levels and biochemical data (enzyme activities) is a recurrent observation in biological systems and might reflect other layers of regulation (Heinemann and Sauer, 2010).

#### Galactose

The profile of Gal consumption was characterized by a plateau (concentration approx. 20 mM), which preceded efficient conversion of Gal to fermentation end-products. This pattern has been described for *L. lactis* when the sugar uptake was exclusively mediated by non-PTS transporters, such as Glc catabolism in a PTS-mutant or Gal catabolism (Castro et al., 2009; Neves et al., 2010). Thus, the *in vivo* NMR data is an additional pointer for the involvement of non-PTS systems in Gal uptake. A plateau was also observed for Glc, but the length was smaller. Interestingly, GlcNAc and Man were used instantly by resting D39 cells, in line with the hypothesis that uptake of these sugars is exclusively mediated by PTS transporters (Bidossi et al., 2012).

The Gal consumption rate was 0.14 ± 0.02 μmol min^−1^ mg^−1^ of protein, a value similar to the one found on Man, and 2-fold lower than the Glc consumption rate (Figure 2C). A similar fold reduction was observed when comparing growth rates on Gal and Man with Glc (Paixão et al., 2015), suggesting that for these two sugars catabolism is major in the multitude of factors determining growth rates. As in growing cells (Paixão et al., 2015), catabolism of Gal in resting cell suspensions showed a pronounced shift to mixed acid fermentation (Figure 2C), with about 35% of the Gal generating acetate (9.8 ± 0.9 mM), ethanol (3.3 ± 0.6mM), and pyruvate (0.6 ± 0.1 mM), while lactate (24.1 ± 0.2 mM) was lower than in the other sugars (Figure 2C).

Yesilkaya et al. (2009) attributed the mixed acid profile of Gal-grown cells of *S. pneumoniae* D39 to the activity of pyruvate formate-lyase (PFL encoded by *pflB*) and pyruvate formate-lyase activating enzyme (encoded by *pflA*). In presence of slow metabolizable sugars and anaerobic conditions PFL competes more efficiently with LDH for pyruvate. In consequence, one more molecule of ATP is generated (via acetate kinase activity), which is certainly an advantage during the metabolism of non-preferential sugars (slow metabolizable). The metabolic shift to mixed-acid fermentation has been the subject of intense research, and the underlying regulatory mechanisms are still under debate. Surprisingly, only the phosphotransacetylase gene (*pta*), was induced by Gal (Table 1, Figure 3C). This expression profile was unexpected, since a pronounced shift to mixed acid fermentation occurs on Gal (Carvalho et al., 2011; Paixão et al., 2015), and induction of the mixed-acid pathways by Gal was reported before (Carvalho et al., 2011). Also, Gal reportedly enhances the activity of pyruvate formate-lyase in *S. mutans* and *L. lactis* (Melchiorsen et al., 2000; Abranches et al., 2008). However, *pflB* appeared as induced by Gal (ratio ~1.90) in *S. pneumoniae* D39 when less restrictive significance criterion (*p* < 0.001 as compared to 0.05/n) was used as in the study by Carvalho et al. (2011), in accordance with regulation at the transcriptional level.

As for many other studies, we fail to observe a complete correlation between expression profiles and pneumococcal phenotypic traits, which ultimately denotes regulation at other cellular layers, such as post-transcriptional and/or metabolic levels.

Resting D39 cells accumulated during the metabolism of [1-^13^C]Gal, FBP and the specific intermediate of the T6P pathway, α-galactose 6-phosphate (α-Gal6P) (Figure 2C). In contrast to growing cells, accumulation of the Leloir intermediates galactose 1-phosphate and glucose 1-phosphate was not observed (Table S5). It is likely that the concentration of these metabolites is below the detection limit of *in vivo* ^13^C-NMR technique. Indeed, the ^31^P-NMR resonances in spectra of Gal-grown cell extracts were relatively weak (Paixão et al., 2015). The pool of FBP accumulated once Gal started to decline, but the maximal concentration was relatively low (approximately 12 mM) (Figure 2C). Reduced FBP levels might derive from the slow flux through glycolysis, and correlate well with the metabolic shift toward mixed-acid fermentation. It is well established that FBP is an activator of LDH, whereas trioses-phosphate (DHAP and GAP), inhibit PFL (Neves et al., 2005). Due to the reversibility of the reactions catalyzed by FBP aldolase and triose 3-phosphate isomerase, low trioses-phosphate concentrations are to be expected when FBP is low. Thus, the activation of LDH and the inhibition of PFL are relieved and a mixed acid profile emerges (Figure 3C). As for other fermentative organisms, the shift toward mixed acid fermentation is multifactorial and involves regulation at the different cellular layers.

The high accumulation of Gal6P in resting cells, also observed in growing cells (Table S5, Figure 2C, Paixão et al., 2015), suggests a metabolic constraint in Gal processing through the tagatose 6-phosphate pathway. In addition, the accumulation of Gal6P is solid evidence for the functionally of a PTS system, since to our knowledge this is the only reaction capable of generating Gal6P in living cells (Neves et al., 2010).

Differently from Man and GlcNAc, Gal induced the expression of Gal putative transporters suggesting their involvement in Gal uptake (Table 1). Upregulation of LacFE was also observed for a different isolate of *S. pneumoniae* D39 on Gal (Carvalho et al., 2011). Of note, this Lac-PTS has been implicated in Gal transport in the closely related organisms *S. mutans* and *L. lactis* (Neves et al., 2010; Zeng et al., 2010). In contrast, the genes SPD_0088-9, which encode the permease proteins of a CUT1 ABC transporter proposed to take up Gal (Bidossi et al., 2012), were downregulated (Table 1 and Table S4). Whether this finding rules out the involvement of the ABC transporter in Gal uptake needs experimental confirmation. It should be noted however, that the evidence previously presented was relatively weak, since inactivation of the transporter resulted only in a mild reduction of Gal utilization (Bidossi et al., 2012). A possible explanation is that the inactivation of the ABC is masked by the activity of other Gal transporters.

Nonetheless, *S. pneumoniae* lacks a high affinity transporter (Paixão et al., 2015), which can also partially explain the inefficient Gal catabolism (Figure 3C).

As expected, Gal induced genes of both the Leloir and tagatose 6-phosphate (T6P) pathways (Table 1, Figure 3C). The duplicated gene *galE-2* was downregulated, underpinning the role of *galE-1* as the functional UDP-glucose 4-epimerase in strain D39. The duplicated gene *galT-1* was not differentially expressed (Figure 3C), strongly pointing to *galT-2* as the Leloir catabolic gene. Interestingly, we have shown that deletion of *galT-2* resulted in *circa* 50 times increased expression of *galT-1*, indicating *galT-1* as a surrogate of *galT-2* (Paixão et al., 2015). Induction of both pathways in response to Gal is a recurrent observation in previous studies from our laboratory (Carvalho et al., 2011; Paixão et al., 2015). Notably, Afzal et al. (2014) observed only increased expression of the T6P pathway genes in response to Gal in *S. pneumoniae* D39. The different results in the two studies most likely derive from differences in cultivation medium and/or other experimental conditions. While we have gathered strong evidence that both pathways are functional, determining the relative contribution of each pathway to the metabolism of Gal is, however, not trivial and would require estimating the flux partitioning between the two routes.

### Catabolism of Glc in galactose-adapted cells

**Figure 4:**
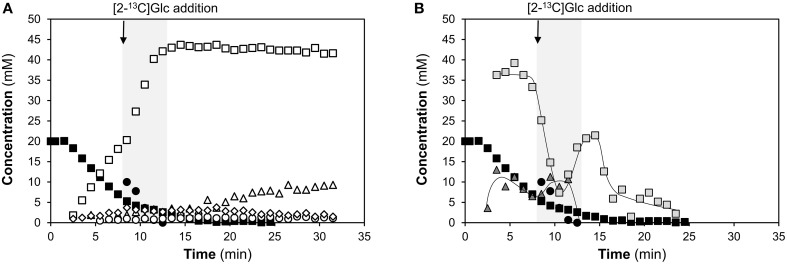
**Metabolism of galactose (Gal), challenged with a pulse of glucose (Glc), using non-growing cells of ***S. pneumoniae*** D39Δ***cps***, monitored by ***in vivo*** NMR**. Kinetics of **(A)** sugar consumption and end-products formation and **(B)** pools of intracellular metabolites. Cells metabolizing a 20 mM pulse of [1-^13^C]Gal were challenged with a 10 mM pulse of [2-^13^C]Glc during the maximal consumption rate of Gal. Time course kinetics were obtained online by *in vivo* ^13^C-NMR. Experiments were carried out at 37°C, under anaerobic conditions and pH control (6.5). Figures are from representative experiments of at least 2 replicates. Lines associated with intracellular metabolite time courses are simple interpolations. The shaded area represents the time spam for which Glc was available, and the arrow the time-point of addition. Symbols: (■), [1-13C]Gal; (●), [2-13C]Glc; (□), total lactate; (▵), acetate (derived from Gal catabolism); (○), ethanol (derived from Gal catabolism); (♢), pyruvate (derived from Gal catabolism); (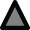), FBP, total fructose 1,6-biphosphate; (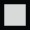) α-Gal6P, α-galactose 6-phosphate derived from Gal catabolism. In this experiment cells were suspended to 24 mg cell dried weight ml^−1^. As compared to the cell suspension in Figure 2C, the biomass is increased by a factor of 1.2, which most likely leads to the reduced Gal plateau in the current situation.

Of the three monosaccharides studied, Gal showed the strongest effect on glycolytic dynamics and end-products profile (Figure 2C). Thus, we asked whether Gal-adapted cells would be able to efficiently catabolize the preferred sugar Glc. To test this hypothesis, cells actively metabolizing 20 mM of [1-^13^C]Gal were challenged with a 10 mM labeled Glc pulse, [2-^13^C]Glc. The kinetics of sugar consumption, end-products, and metabolic intermediates is depicted in Figure 4, as monitored by *in vivo* ^13^C-NMR.

The addition of Glc decreased the rate of Gal utilization 3.5-fold in comparison with the initial Gal consumption rate (0.14 ± 0.02 μmol min^−1^ mg^−1^ of protein), showing a preference for Glc over Gal. This result is in agreement with that showing that Gal transport is inhibited by Glc (Fleming et al., 2015). Although Gal consumption was hindered by the presence of Glc, *S. pneumoniae* was able to catabolize both substrates simultaneously (Figure 4).

Glc was readily consumed as soon as it became available, at a consumption rate (0.27 ± 0.01 μmol min^−1^ mg^−1^ of protein) similar to that of Glc as a sole substrate (Figure 2D). This behavior shows that Gal-adapted cells are apt to efficiently metabolize Glc. Indeed, no major changes in transcription of glycolytic genes or *manLMN* (PTS-Man) were observed on Gal as compared to Glc.

The rate of lactate production doubled immediately after the pulse of Glc, shifting the metabolism to a more homolactic profile (Figure 4A). Lactate (43 ± 1.8 mM) accounted for 71% of the total substrate (Glc and Gal) consumed, as compared to 60% on Gal alone. This value is in good agreement with the estimated (68.2%) from a 2:1 Gal to Glc ratio and the 60% and 85% sole conversions of Gal and Glc, respectively. Of the total lactate, about 37% was labeled on C_2_, and thereby derived from [2-^13^C]Glc. Other products from this substrate were acetate (2.6 ± 0.4 mM) and pyruvate (0.8 ± 0.3 mM). The mixed acid products (acetate and ethanol) decreased from 33% (Gal alone) to 22% of the total substrate. Moreover, pyruvate accumulated to a maximal concentration 5.5-fold higher than those on Gal alone. This behavior suggests a bottleneck downstream of pyruvate. Curiously, the negative effect of Glc on mixed acid products was more pronounced on ethanol than acetate.

Addition of Glc, caused a sudden drop on the pool of α-Gal6P and rise of the FBP level, a trend that favors lactate production (Figure 4B). All in all, these results show that Glc exerts a negative effect (metabolic inhibition) on the mixed acid fermentation profile.

### Effect of glucose on the growth of *S. pneumoniae*

**Figure 5:**
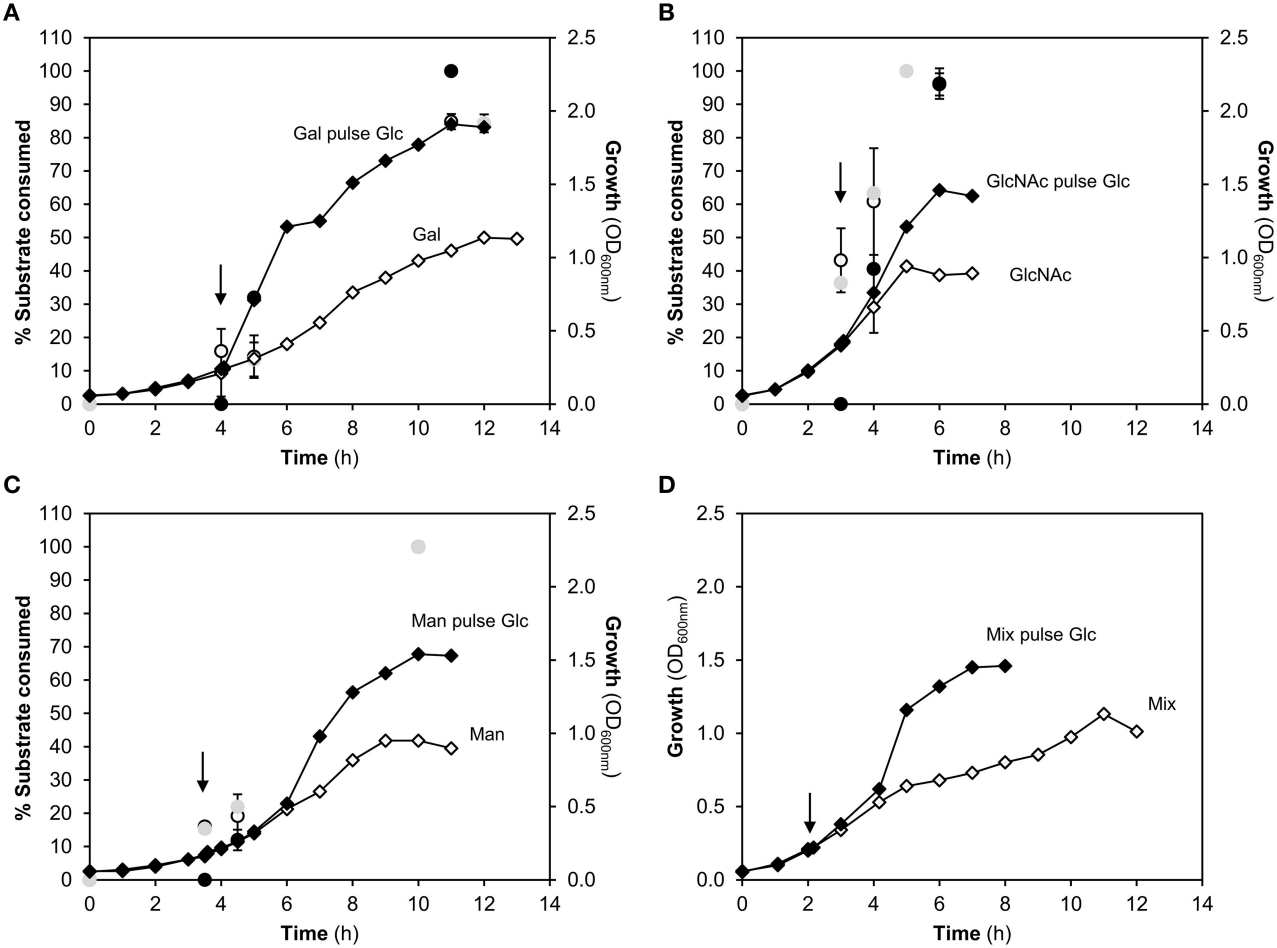
**Representative growth profiles of ***S. pneumoniae*** D39 in presence of different carbon sources challenged or unchallenged with a pulse of glucose (Glc) and percentage of substrate consumed**. Growths were conducted in CDM supplemented with: **(A)** galactose (Gal); **(B)** N-aceylglucosamine (GlcNAc); **(C)** mannose (Man), and **(D)** a mixture of Gal, Man, and GlcNAc, challenged or unchallenged with a 10 mM Glc pulse given at mid-exponential phase of growth. Cells were grown at 37°C, under semi-anaerobic conditions, without pH control (initial pH 6.5). The black arrow indicates the time of the Glc pulse. The profiles are averages of two independent growths (unless stated otherwise) and standard deviation is depicted. The percentages of GlcNAc and Man consumed without the Glc challenge correspond to only one representative growth. Symbols: (●), percentage of Glc consumed in pulse chase experiments; (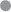), percentage of Gal, GlcNAc, or Man consumed in unchallenged cells; (○), percentage of Gal, GlcNAc, or Man consumed in Glc challenged cells; (♦), represent the growth curve of cells growing on glycan-derived sugars challenged with a glucose pulse at mid-exponential phase of growth; (♢), represent the growth curve of cells growing on glycan-derived sugars unchallenged with a glucose pulse.

The significant impact of Glc over Gal catabolism at the metabolic level was evident on resting cells of *S. pneumoniae*. Thus, we deemed important to assess the impact of Glc during growth of pneumococcus on the three glycan-derived monosaccharides. To this end, Glc was added to exponential cells growing on Gal, Man, GlcNAc or a mixture thereof (Figure 5).

For all the conditions tested, an increase of growth rate was observed upon Glc addition (Figure 5, Table 3). This positive effect was more pronounced in Gal-adapted cells (Figure 5A, Table 3), for which the final biomass and the maximal specific growth rates were increased by 74 and 172%, respectively. Furthermore, Glc induced biphasic growth, with a phase displaying a maximal growth rate of 0.87 ± 0.07 h^−1^), followed by slower growth (0.14 ± 0.01 h^−1^). In the sugar mix, the final biomass increased by 30%, while the time to reach these maximal values was shortened by 43% (Figure 5D).

**Table 3:** **Growth and energetic parameters, carbon and redox balances and substrate consumed, obtained for ***S. pneumoniae*** D39 grown on CDM in the presence of N-acetylglucosamine (GlcNAc), galactose (Gal), mannose (Man) or in a mixture of sugar (GlcNAc, Man, and Gal) challenged or not with a 10 mM glucose (Glc) pulse given at mid-exponential phase of growth**.

	**GlcNAc^a^**	**GlcNAc pulseGlc**	**Gal^a^**	**Gal pulseGlc**	**Man^a^**	**Man pulseGlc**	**Mix^a^**	**Mix pulseGlc**
[substrate]_initial_ (mM)	12.3±0.6	11.7±0.0	13.4±1.3	12.0±0.0	12.4±0.1	14.1±0.4	6.5±0.3^e^	6.1±0.8^e^
Product yields								
Lactate	1.58±0.04	1.66±0.01	0.04±0.01	1.14±0.07	1.43±0.12	1.42±0.06	1.25±0.02	1.811^c^
Formate	0.15±0.07	0.05±0.01	1.47±0.18	0.50±0.01	0.31±0.04	0.15±0.01	0.36±0.06	0.09^c^
Acetate	0.06±0.01	ND	0.75±0.13	0.27±0.05	0.20±0.02	0.08±0.00	0.15±0.01	0.03^c^
Ethanol	0.03±0.01	0.01±0.01	0.72±0.05	0.24±0.04	0.14±0.04	0.06±0.00	0.18±0.03	0.02^c^
μ_max_ (h^−1^)^b^	0.40±0.04	0.44±0.13	0.32±0.04	0.87±0.07^d^	0.37±0.06	0.46±0.06	0.54±0.15	0.67±0.19
OD_600max_	0.99±0.08	1.35±0.17	1.12±0.26	1.95±0.07	1.03±0.08	1.59±0.07	1.19±0.11	1.55±0.09
Substrate Consumed (%)	100±0	96±4	84±7	92±1	100±0	100±0	86±2	79^c^
Substrate Recovery	84±3	86±0	76±9	83±4	89±3	78±3	79±3	95^c^
Redox Balance	83±3	84±1	75±5	82±1	85±3	77±2	81±4	93^c^
ATP yield (mol mol^−1^ substrate)	1.74±0.08	1.67±0.01	2.27±0.31	1.93±0.14	1.98±0.04	1.65±0.06	1.73±0.06	1.89^c^
YATP (g biomass mol^−1^ATP)	16.8±0.9	16.7±0.3	14.9±0.5	18.7±0.5	16.2±0.4	14.6±0.2	16.4±0.4	14.7^c^
Ybiomass (g mol^−1^ substrate)	29.3±2.9	27.9±0.7	34.1±5.8	36.0±1.6	31.9±0.1	24.0±0.6	28.3±1.8	27.7^c^

*The controls (unchallenged cells) are displayed as reported (Paixão et al., 2015). Growth experiments were made at 37°C without pH control (initial pH 6.5). Values represent the average and standard deviation of at least two independent growth experiments and were estimated at the time point of growth arrest (maximal biomass)*.

a *Values as reported by Paixão et al. (2015), except growth rate*.

b *Maximal specific growth rate determined in the time range after the Glc pulse*.

c *Values of a representative experiment*.

d *Maximal specific growth rate. μ_2_ = 0.14±0.01 h^−1^*.

e *The value represents the average ± standard deviation of the concentration for all the sugars separately. Individual averages for sugar mixes are: Gal, 6.4 ± 0.7 mM; Man, 6.6 ± 0.1 mM; and GlcNAc, 6.7 ± 0.2 mM*.

*Individual averages for sugar mixes with pulse of Glc are: Gal, 5.7 ± 0.1 mM; Man, 5.9 ± 1.4 mM; and GlcNAc, 6.5 ± 0.4 mM*.

*ND, Not detected as product of pyruvate metabolism*.

*Substrate recovery is the percentage of carbon in metabolized sugar that is recovered in the fermentation products (lactate, ethanol, acetate, and formate)*.

*Redox balance is the ratio between [lactate] + 2 × [ethanol] and 2 × [substrate consumed] multiplied by 100*.

Independently of the carbon source in the medium, growing *S. pneumoniae* cells consumed Glc at once after addition (Figure 5). A similar behavior was described for the resting cells (Figure 4). A lag phase, typical of the diauxic behavior associated with adaptation to the additional substrate, was not observed for any of the conditions tested. The rate of Glc utilization was higher than that of the other sugars, as evidenced by the higher quantity of Glc processed over a defined period of time. In summary, *S. pneumoniae* is well equipped to use its preferred substrate Glc, regardless of pre-conditioning to other sugars. However, utilization of Glc does not exclude co-metabolism of the other sugars.

### Glucose addition represses mixed acid fermentation profile

In resting cells actively metabolizing Gal, Glc promoted a more homolactic fermentation profile. The effects of Glc addition to the end-products formed during growth are shown on Table 3. Cells exponentially growing on Gal, GlcNAc or the sugar mix, when challenged with Glc displayed increased lactate yield, which was condition dependent (Table 3). Curiously, this pattern was not observed for Man-adapted cells (Table 3). Gal-adapted cells showed the largest change in lactate yield, even though no induction of *ldh* was observed (Table S6).

Furthermore, Glc addition repressed the formation of mixed acid fermentation products (acetate, ethanol, and formate) to a larger extent than the positive effect on lactate production in all the conditions tested, except for Gal (Table 3). Generally, Glc repressed the expression of genes involved in mixed acid fermentation (SPD_1834, *pflB, pta*) (Table S6), and the reduced transcription positively correlates with the reduced levels of mixed-acid products (Table 3). On Man, however, the addition of Glc resulted in elevated levels of *pflB, pta* and SPD_1834 transcripts, but still the mixed acid products were lessened (Table 3, Table S6). These results suggest that under this condition, regulatory mechanisms other than transcription are present.

Based on the results with resting cells we proposed that the partitioning between lactate and mixed acid products is largely regulated at the metabolic level. Overall, the results with growing cells further strengthen this view, without ruling out regulation at the transcriptional level.

### Effect of a glucose pulse on the expression of genes involved in sugar metabolism

The effect of a Glc challenge to cells growing on the glycan-derived sugars was assessed at the transcriptional level (Table S6). The number of genes significantly differentially expressed in response to Glc was dependent on the sugar (Tables S7–S10). The largest transcriptional response was observed in Man-adapted cells (434 genes out of 1738) and the smallest in cells adapted to a sugar mixture (5.5% of the total transcripts) (Figure S1B). In Gal-adapted cells, 249 genes were regulated by Glc, whereas in GlcNAc-adapted cultures 111 genes were differentially expressed after the Glc pulse (Figure S1B). The transcriptional response of Gal and Man-adapted cells to Glc included altered expression of 157 shared genes, of which 101 were exclusive of these conditions. Despite the large number of common genes in the two conditions, the direction of regulation (upregulation vs. downregulation) of these genes was not necessarily the same. Indeed, Glc exerted positive (upregulation) and negative (downregulation) regulation in all conditions tested. Gal-adapted cells showed the highest number of upregulated genes (53% of the total), whereas GlcNAc-adapted cells showed the lowest (22.5% of the total) (Tables S7, S8), an indication that Glc is a mightier repressor of Gal catabolism than of the catabolism of the other sugars. Interestingly, the lesser response in the sugar mix denotes better capacity of these cells to cope with a Glc stimulus, which can be perceived as improved metabolic fitness.

The results presented in Figure S1B and Table S6 clearly show sugar-dependent responses to a Glc stimulus. Progression from colonization to invasive disease is presumably associated with a change from an environment nearly devoid of Glc (nasopharynx) to niches rich in this sugar (blood, inflamed lung). Thus, determination of the transcriptional responses to Glc in clinical serotypes of *S. pneumoniae* should be pursued in the future.

For all the conditions tested, the COG category of “carbohydrate metabolism and transport” (G) was overrepresented (Table S11), with the proportion of genes showing altered expression in response to Glc as follows: 32.4, 28.1, 16.9, and 12.4% of the total transcripts on GlcNAc, sugar mix, Gal, and Man, respectively. Also, most of the genes belonging to category G were downregulated by Glc, except for Man-adapted cultures in which a higher number was induced.

Glucose addition in GlcNAc-adapted cells repressed the expression of putative and proven transporters of this amino sugar (Paixão et al., 2015, Table S6), among which the PTS-Man SPD_0262-3-4 (*manLMN*) was downregulated. This system is most likely the major GlcNAc transporter in *S. pneumoniae* D39 (Bidossi et al., 2012). In addition, is also the principal Glc uptake transporter in *S. pneumoniae* (Bidossi et al., 2012). Downregulation of glucose transport proteins in response to Glc is not without precedent, and in *E. coli* is one of the molecular mechanisms in the cellular response to prevent phosphosugar stress (Vanderpool, 2007).

Genes involved in glycolysis were not differentially expressed upon Glc addition. Interestingly, Glc repressed the expression of the dedicated GlcNAc-specific catabolic gene *nagB*, but not *nagA* in the same transcriptional unit. Furthermore, only *nagB* was found upregulated in GlcNAc-growth medium (using a *p* < 0.001) as compared to Glc. Thus, each gene in the *nagAB* operon is subjected to specific regulation.

The direction of the regulation elicited by Glc on Man-adapted cells was in most opposite to that observed in the other conditions studied (Table S6). While an explanation cannot be put forward, one can speculate that the Man signaling cascade is heavily intertwined with that of Glc, as both sugars are taken up by the Glc/Man-PTS, a well-recognized major player in catabolite control in low-GC Gram-positive bacteria. How the cell discriminates between the two sugars is unknown. From our studies it is clear that the cellular responses to Man (transcriptional and physiological/metabolic) are large and unique, but the underlying molecular mechanisms remain to be elucidated. For example, Glc induced the expression of transporters presumably involved in Man uptake (Table S6) as well as the expression of the glycolytic genes *gap* and *eno* (Table S6), but did not alter *manA* transcription.

We have shown that Glc hinders the use of Gal (Figure 4) and this observation is fully substantiated by the transcriptional response to Glc in Gal-adapted cells. In Gal-adapted cells Glc downregulated the transporter genes SPD_0559-0-1 and *lacEF-2* (Lac-family PTS) (Table S6), which together with the observed upregulation on Gal as compared to Glc-grown cells (Table 1, Table S6), point toward their involvement on Gal uptake. The latter transporter is found in operon-II of the *lac* gene cluster, which comprises also a 6-phospho β-galactosidase, *lacG-2* (SPD_1046), and the transcriptional activator *lacT* (SPD_ 1049) (Afzal et al., 2014). While *lacG-2* was not differentially expressed, *lacT* was downregulated by Glc (Table S8), and so was operon-I, encoding the T6P pathway (*lacABCD*). Moreover, Glc downregulated the Leloir genes *galk* and *galT-2*, as well as the transcriptional regulator *galR* (Table S6). Our results provide strong evidence supporting the repressing effect of Glc over the catabolism of Gal. Previously, Carvalho et al. (2011) showed that while CcpA repressed the Leloir genes independently of the carbon source, Gal was an effective inducer of this pathway. On the other hand the T6P pathway was weakly repressed by CcpA on Glc and activated on Gal. In line, Afzal et al. (2014) could not observe a regulatory effect of CcpA on the *lac* gene cluster. Instead, LacR was the transcriptional repressor of the *lac* operon-I (T6P pathway) on Glc, but not of operon-II, which was under the control of LacT.

For the sugar mix, a negative effect over genes encoding GlcNAc, Man and Gal transporters was observed (Table S6). Furthermore, Glc downregulated genes in Gal catabolic pathways (*lacAB, galk and galT-2*), but had no impact on the expression of genes devoted to the catabolism of GlcNAc and Man.

### The expression profile of virulence factors displays a sugar dependency

It is now well accepted that sugar metabolism and virulence are strongly connected, thus contributing in different ways to the pathogenesis of the pneumococcus (Iyer and Camilli, 2007; Shelburne et al., 2008). Therefore, we investigated the influence of the carbohydrates GlcNAc, Man and Gal on the expression of known virulence factors (Table S1, Table 4). How these virulence factors responded to a Glc stimulus during growth on the different sugars or a sugar mix was also examined (Table 4).

**Table 4:** **Significantly differentially expressed virulence factors (up- or downregulated) of cells of ***S. pneumoniae*** D39 grown in CDM supplemented with N-acetylglucosamine (GlcNAc), mannose (Man), or galactose (Gal) as compared to glucose (Glc) grown cells, or adapted to these single sugars or to a mixture thereof challenged with a Glc pulse and compared to unchallenged cells, determined by DNA microarrays^a^^,^^b^**.

**Locus_tag**	**Gene**	**Product**	**Up- or downregulation^c^**						
			**GlcNAc**		**Man**		**Gal**		**Mix^d^**
			**without Glc**	**with Glc**	**without Glc**	**with Glc**	**without Glc**	**with Glc**	**with Glc**
SPD_0063	*strH*	beta-N-acetylhexosaminidase	0.43		0.90	1.11			−0.48
SPD_0065	*bgaC*	Beta-galactosidase 3		−0.81		0.64			−1.38
SPD_0126	*pspA*	pneumococcal surface protein A			0.68		0.76	−0.79	−0.50
SPD_0250		pullulanase, extracellular			0.47	1.00	−0.70		
SPD_0373		hypothetical protein SPD_0373		−1.10		0.76	1.43	−1.50	−1.30
SPD_0420	*pflB*	pyruvate formate-lyase		−0.53	1.25	0.70			−0.99
SPD_0558	*prtA*	cell wall-associated serine protease PrtA				0.47	−0.41		
SPD_0562	*bgaA*	beta-galactosidase precursor, putative		−0.33		0.71	1.61	−0.82	
SPD_0634		hypothetical protein SPD_0634	−0.89		−1.24				
SPD_0635		cation-transporting ATPase, E1-E2 family protein			−1.23				
SPD_0667	*sodA*	superoxide dismutase, manganese-dependent						−0.47	
SPD_0701	*ciaR*	DNA-binding response regulator CiaR	0.82		0.57	−0.53			
SPD_0729		hemolysin-related protein			0.44	0.48			
SPD_0853	*lytB*	endo-beta-N-acetylglucosaminidase precursor, putative	0.82						
SPD_0889	*phtD*	pneumococcal histidine triad protein D precursor				0.40			
SPD_1012	*eno*	enolase			0.61	0.33			
SPD_1018	*iga*	immunoglobulin A1 protease precursor	−0.30		0.49	0.48	0.72	−0.63	
SPD_1050	*lacD*	tagatose 1,6-diphosphate aldolase					3.00	−2.02	
SPD_1384		cation efflux family protein				−0.58		0.44	
SPD_1409		sugar ABC transporter, ATP-binding protein		−0.81		0.86	−0.52		−0.68
SPD_1464	*tpx*	thiol peroxidase					0.80	−0.64	−0.47
SPD_1634	*galK*	galactokinase			0.64	0.77	3.30	−1.69	−0.89
SPD_1636		alcohol dehydrogenase, zinc-containing			−0.62				0.69
SPD_1652		iron-compound ABC transporter, iron-compound-binding protein	−0.47			−0.58	−1.16	0.97	
SPD_1726	*ply*	pneumolysin	−0.36		−0.56				
SPD_1774	*pflA*	pyruvate formate-lyase activating enzyme		−0.67		0.73			−0.48
SPD_1797	*ccpA*	catabolite control protein A		−0.60		0.56			
SPD_1823	*gap*	glyceraldehyde-3-phosphate dehydrogenase			0.53	0.39			
SPD_1965	*pcpA*	choline binding protein PcpA				0.28		0.50	
SPD_2068		serine protease	1.06		0.73	−0.92	0.68		

a *Subset table of Tables S2–S4, S7–S10*.

b *As reviewed in Table S1*.

c *Values of ln-ratio. Positive values indicate upregulation and negative values indicate downregulation*.

d *Mixture of Gal, Man, and GlcNAc*.

A clustering analysis identified only two virulence genes as differentially regulated in the three sugars tested, showing that induction of virulence determinants is sugar dependent (Table 4, Figure S3). The immunoglobulin A1 protease precursor (SPD_1018, *iga*) (Poulsen et al., 1996) was upregulated in Gal- and Man-containing medium and downregulated in presence of GlcNAc, whereas the serine protease (SPD_2068) (Sebert et al., 2002) gene was upregulated in all growth conditions. Interestingly, Man also induced the expression of DNA-binding response regulator *ciaR*, which was shown to regulate the serine protease. Mutants in both genes show reduced virulence, but in the Δ*ciaR* is most likely due to downregulation of the serine protease (Ibrahim et al., 2004). The effect of Glc on the expression of virulence genes was also sugar dependent (Table 4). A clustering analysis showed that a single virulence gene was regulated by Glc in all the conditions studied (Table S12, Figure S2). This gene, SPD_0373, which is found in an operon associated to virulence in bacteraemia and pneumonia (Paterson et al., 2006) was downregulated by Glc, except for Man-adapted cells.

Galactose influenced the expression of the largest pool of virulence genes (8.2% of the significantly differential expressed genes), of which 5.5% were upregulated (Table 4). Of note, the virulence factor β-galactosidase (*bgaA*) was highly induced (Table 4). Growing evidence supports the role of this gene in early stages of host-pathogen interactions (Song et al., 2008; Limoli et al., 2011). Moreover, the catabolic genes *lacD* and *galK* were upregulated. We have shown that *lacD* and *galK* mutants are impaired in their ability to colonize the nasopharynx and display attenuated virulence in a respiratory infection murine model, after intranasal challenge (Paixão et al., 2015). Our earlier assumption that Gal metabolism is linked to virulence (Paixão et al., 2015) is further strengthened by the transcriptome data. Interestingly, the expression of *bgaA, lacD* and *galK* was downregulated in Gal-adapted cells by Glc (Table 4). Glc induced the expression of the choline binding protein (*pcpA*), a protein involved in the adherence to nasopharyngeal and lung epithelial cells (Khan et al., 2012), and virulence determinant in mouse models of pneumonia and sepsis (Johnston et al., 2006; Glover et al., 2008).

In GlcNAc-grown cells there was upregulation of *strH*, encoding the N-acetylglucosaminidase (Table 4). The removal of terminal GlcNAc by StrH from human N-glycans has been implicated in pneumococcal colonization and pathogenesis, since it can facilitate sugars (GlcNAc) for growth, but it might also promote resistance to opsonophagocytic killing by avoiding complement deposition (King et al., 2006; Burnaugh et al., 2008; Dalia et al., 2010). Interestingly, the gene was not differently regulated in Glc challenged GlcNAc-adapted cells. In this sugar, all virulence genes responding to the Glc stimulus were downregulated (Table 4). Curiously, Glc stimulus repressed genes common to two sugar-adapted conditions (*bgaC, bgaA, pflB, pflA, galK*) (Table 4). This observation could indicate that while expressed in colonization states (glycans as carbon sources), these genes are negatively regulated in conditions where Glc is the predominant sugar, like the blood or inflamed lungs. Thus it is tempting to suggest that while important for colonization, those functions are not required in invasive disease.

Induction of *pflB* and the glycolytic genes *eno* and *gap* during growth on Man (Table 4) is consistent with their roles in early phases of infection, since Man exists in gycoconjugates. *pflB*, was recently found to contribute to attenuated colonization of the nasopharynx and lungs and delayed bacteraemia in mice infected intranasally (Yesilkaya et al., 2009), whereas *eno* and *gap* were shown to bind to plasminogen and plasmin, and hence can be of importance for the dissemination of the pathogen through host tissues (Bergmann et al., 2001, 2004). These genes were still upregulated when Man-adapted cells were spiked with Glc, but the fold-expression values were smaller.

In *S. pneumoniae* cells growing on a sugar mix, Glc exerted mainly negative regulation (9 out of 10 virulence genes differently regulated were repressed by Glc). The gene encoding the zinc-containing alcohol dehydrogenase (SPD_1636) was, however, upregulated (Table 4). Virulence studies revealed that this gene is implied in survival in the bloodstream (Stroeher et al., 2007).

Of all the conditions tested, the smallest transcriptional response elicited by Glc was for cells adapted to the sugar mix (Tables S7–S10). Curiously, it was in this condition that the highest fraction of virulence genes influenced by Glc was found (10.4% of the significantly differentially expressed genes) in comparison to 6.3, 4.8, and 4.4% in GlcNAc-, Man- and Gal-adapted cells, respectively. This result strengthens our hypothesis that cells growing on the sugar mix are better equipped to cope with ever-changing environments. In these cells, a major adaptation is apparently the downregulation of virulence factors important in colonization niches. Of note, most of the virulence genes were negatively regulated by a Glc stimulus except in Man-adapted cells for which a significant number of virulence genes were upregulated. The *S. pneumoniae* physiological responses to Man are far from being understood and should be the focus of future research. In light of the transcriptional response to a Glc stimulus during growth on Gal, GlcNAc and the sugar mix, we can speculate that the development of pneumococcal virulence traits occurs in the ecological niche (nasopharynx), during colonization, where Gal, Man, and GlcNAc are prevalent in comparison to free Glc. Disease, on the other hand is accidental, as it culminates in a dead-end for the colonizing microorganism, and likely occurs from an imbalance between the host and the microbe. In the disease state, the bacterium represses functions essential for colonization, but no longer needed when Glc is the predominant substrate for growth. In accordance with this hypothesis, we have shown that pneumococcal mutants in the Gal catabolic genes administrated intravenously were not attenuated in murine models of disease (Paixão et al., 2015).

Overall, our results show that sugars influence the virulence potential of *S. pneumoniae* D39 both by modulating the expression of specific catabolic pathways (*in vivo* fitness) as well as the expression of virulence factors (Table 4). Similar findings have been reported for other *Streptococcaceae*, such as *S. mutans* and *S. suis* (Ferrando et al., 2014; Moye et al., 2014b). Indeed, successful infections rely on colonization, multiplication and transmission to a new host, and therefore the line between factors required for growth and virulence determinants is blurred (Hava et al., 2003; Weiser, 2010).

## Conclusions

In this work we have conducted a systems approach to evaluate the *S. pneumoniae* response to sugar availability. The combined transcriptional, physiological, and metabolic data collected revealed a strong carbohydrate-dependency on the phenotypic traits of *S. pneumoniae*. Despite the relatively simple metabolism of *S. pneumoniae*, which processes sugars through the Embden-Meyerhof-Parnas pathway to pyruvate, the transcriptional and metabolic responses elicited by each monosaccharide are remarkable and specific. This is especially relevant considering that generally this bacterium resides in the human nasopharynx, an environment poor in Glc, but rich in glycans. But during progression to disease and in disease states *S. pneumoniae* is subjected to changing environments that presumably are enriched in Glc. Our results firmly show that Glc is a preferential substrate for growth of *S. pneumoniae* and while growing on other sugars the bacterium avidly uses Glc when available. The specific response to the Glc stimulus results in changes both at the metabolic level (Figures 4, 5) and in gene regulation (Table S6), which allow for short and long term adaptation. Interestingly, cells adapted for growth on a sugar mixture displayed the smallest transcriptional response to Glc, suggesting improved resilience of *S. pneumoniae* when exposed to a multitude of sugars. In the human nasopharynx, *S. pneumoniae* is exposed to a fluctuating nutritional milieu that results from a fragile balance between varied factors (host, microbiota, environmental stimulus). In addition, deglycosylation of human glycans by bacterial glycosidases generates a varied sugar mixture. In the context of our observations, exposure to such a diverse environment improves the fitness of *S. pneumoniae*.

Carbohydrates specifically modulate the expression of virulence genes, thus influencing the virulence potential of the pneumococcus. We suggest that the nasopharynx is the reservoir for the development of niche-specific virulence traits, essential for successful colonization of the niche. Most of these virulence factors are downregulated by a Glc stimulus, and are therefore not required in disease. Collectively, our data strengthens the link between sugar metabolism and virulence. Indeed, effective infections rely on colonization, multiplication and transmission to a new host, and therefore factors required for growth are also virulence determinants (Hava et al., 2003; Weiser, 2010).

The “omic” data collected at different regulatory layers can in the future be used to fuel multi-scale mathematical models. Such mathematical representation of metabolism hopefully will contribute to deepen our understanding of how a functional state arises from the components, and ultimately will facilitate the identification of novel targets for alternative therapeutic and preventive drugs.

## Author contributions

LP and AN designed the experiments. LP performed all the experiments, except the microarrays experiment which was performed by TK. LP and AN analysed the data. JC, TK, and LP analysed the microarrays results. OK and SV contributed to the critical reading and supplying of materials. All authors contributed to the critical reading and writing of the manuscript.

### Conflict of interest statement

The authors declare that the research was conducted in the absence of any commercial or financial relationships that could be construed as a potential conflict of interest.
