# Supplementary material for: Transcriptional and metabolic effects of glucose on Streptococcus pneumoniae sugar metabolism
Source: Front Microbiol. 2015 Oct 7;6:1041. doi: 10.3389/fmicb.2015.01041 (PMC4595796; doi:10.3389/fmicb.2015.01041)
Supplement: Supplementary file 14 [file DataSheet1.DOCX]

***Supplementary Material***

**Transcriptional and metabolic effects of glucose on**

***S. pneumoniae* sugar metabolism**

**Laura Paixão, José Caldas, Tomas G. Kloosterman, Oscar P. Kuipers, Susana Vinga and Ana Rute Neves ***

*** Correspondence:** Dr Laura Paixão: lpaixao@itqb.unl.pt

1. **Supplementary Data**
   1. **Supplementary Text 1**

**Detailed description of the microarrays results of pneumococcal cells grown on the glycan-derived sugars: Gal, Man and GlcNAc**

The number of genes significantly differentially expressed was sugar specific (Figure S1). Mannose elicited the largest transcriptional response, with a total of 247 genes out of 1738 (about 14.2%) showing altered mRNA levels as compared to Glc. In opposition, Gal-grown cells displayed the lowest percentage of significantly differentially expressed genes (8.4%) in comparison with Glc.

A total of 198 genes were significantly differentially expressed in cells grown in GlcNAc-containing medium (11.4%).

Man elicited a global effect and altered the expression of genes in glycolysis and pyruvate metabolism, as opposed to GlcNAc and Gal.

On Gal 44.5% of the differentially expressed genes, were upregulated, whereas on GlcNAc and Man, the fraction of induced genes was 37.4% and 40.9%, respectively, lower values than on Gal. In addition, the expression ratios were largest on Gal (Tables S2, S3 and S4), suggesting that Glc is a stronger repressor of Gal metabolism than metabolism of Man or GlcNAc.

Interestingly, only a small percentage (1.8%) of genes significantly differentially expressed was common to the three carbon sources (Figure S1A and Table S13). Of these, 12% were upregulated in the presence of the glycan-derived sugar, whereas the majority was seemingly activated by Glc. This was indeed the case for the SPD_0277-8-9-0-1-2-3 locus comprising the Lac-PTS transporter *celBCD*, a 6-phospho-beta-glucosidase *celA*, a transcriptional regulator and two hypothetical proteins, which was downregulated on all conditions tested. Also downregulated was SPD_0502, which encodes the IIABC components of a Glc-family PTS system (Table S13). Thus, expression of two sugar transporters, SPD_0502 and CelBCD, supposedly involved in the translocation of beta-glucosides (Cote *et al.*, 2000; McKessar and Hakenbeck, 2007; Bidossi *et al.*, 2012) is induced by Glc, suggesting a possible role of these transporters in Glc internalization. The involvement of transporters other than the PTS-Man (ManLMN) on Glc transport has been postulated, but not proven to date (Bidossi *et al.*, 2012).

Among the three sugars, GlcNAc and Man showed the highest number of differentially expressed genes in common (46 genes), while Gal and GlcNAc had the least number of common differentially expressed genes (only 13 genes) (Figure S1A). In conclusion, growth on Man and GlcNAc showed the lowest differential regulation at the transcriptional level, thus the simultaneous utilization of GlcNAc and Man is likely more favourable than of one of the previous together with Gal.

Overrepresentation of COG categories among the significantly differentially expressed genes displayed a relation to the sugar used (Table S11). In Gal-grown cells the COG category of “carbohydrate metabolism and transport” (G) was overrepresented (28 genes out of 146, 15 of which were induced) (Tables S4 and S11). On GlcNAc, the “amino acid metabolism and transport” (E) and the “translation, ribosomal structure and biogenesis” (J) categories showed the highest number of genes significantly, differentially expressed (18.7% and 14.6%, respectively). While in the E category 8 genes out of 37 were induced, only one gene belonging to the J category was upregulated (Tables S2 and S11). In Man-grown cells no differential COG categories were overrepresented, according to the criterion established (Tables S3 and S11). In accordance, Man-grown cells displayed the highest percentage (26.3%) of genes encoding hypothetical proteins differentially expressed.

**References**

Bidossi, A., Mulas, L., Decorosi, F., Colomba, L., Ricci, S., Pozzi, G., *et al.* (2012) A functional genomics approach to establish the complement of carbohydrate transporters in *Streptococcus pneumoniae*. *PLoS ONE* **7**: e33320.

Cote, C.K., Cvitkovitch, D., Bleiweis, A.S., and Honeyman, A.L. (2000) A novel beta-glucoside-specific PTS locus from *Streptococcus mutans* that is not inhibited by glucose. *Microbiol Read Engl* 146 ( Pt 7): 1555–1563.

McKessar, S.J., and Hakenbeck, R. (2007) The two-component regulatory system TCS08 is involved in cellobiose metabolism of *Streptococcus pneumoniae* R6. *J Bacteriol* **189**: 1342–1350.

Paixão, L., Oliveira, J., Veríssimo, A., Vinga, S., Lourenço, E.C., Ventura, M.R., *et al.* (2015) Host glycan sugar-specific pathways in *Streptococcus pneumoniae*: galactose as a key sugar in colonisation and infection. *PLOS ONE* **10**: e0121042.

1. **Supplementary Figures and Tables**

## Supplementary Figures

**
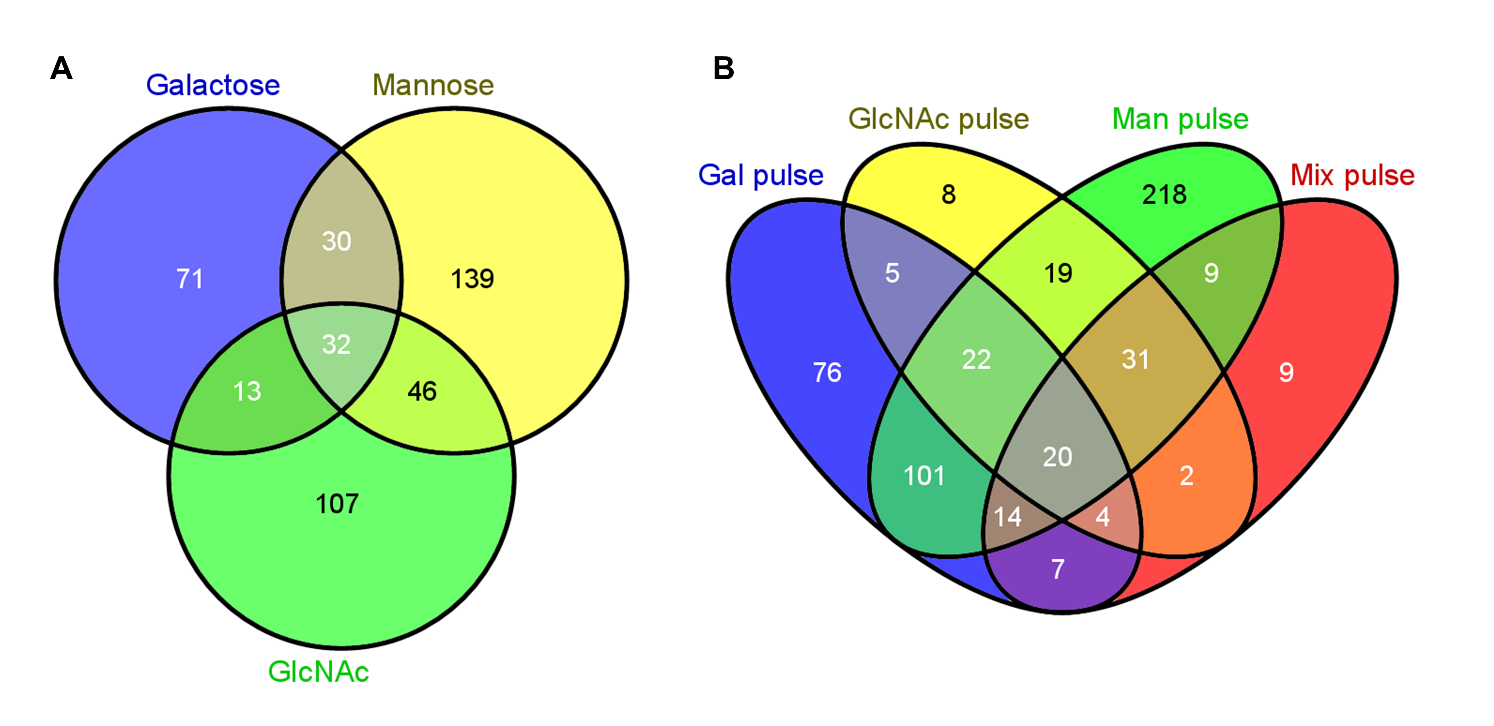
**

**Figure S1. Comparison of the genome-wide transcriptional response to different carbohydrate availabilities.** Venn diagrams of the significantly differentially expressed genes during growth on (**A**) galactose (Gal), mannose (Man) and N-acetylglucosamine (GlcNAc) and (**B**) adapted to grow on galactose, mannose, N-acetylglucosamine or in a mixture thereof (Mix) and submitted to a pulse of Glc. Figures were generated using VENNY (http://bioinfogp.cnb.csic.es/tools/venny/index.html).

**
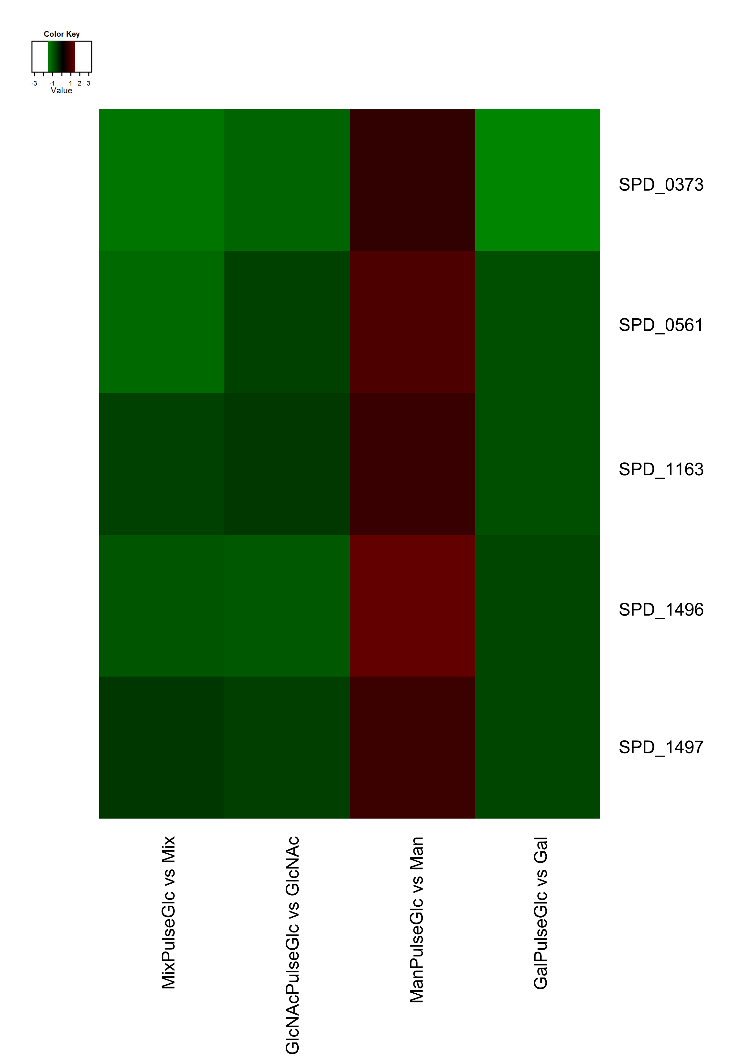
**

**Figure S2.** **Visual representation of the clustering analysis performed for monosaccharides challenged with a pulse of glucose *versus* unchallenged cells, targeting the selected categories of genes.** Colour scale: ln-ratio of expression. Red, upregulated gene in the transcriptome analysis; green, downregulated gene in the transcriptome analysis. The figure was generated targeting the gene categories of interest (glycolysis, pyruvate metabolism, sugar dedicated transporters and catabolic genes as reviewed by Paixão *et al.*, (2015) and classical virulence factors). According to NCBI annotation: SPD_0373, hypothetical protein; SPD_0561, PTS system transporter subunit IIC; SPD_1163, N-acetylneuraminate lyase; SPD_1496, PTS system transporter subunit IIBC; SPD_1497, N-acetylmannosamine-6-phosphate 2-epimerase (*nanE-1*). Glc, glucose; Man, mannose; Gal, galactose; GlcNAc, N-acetylglucosamine; Mix, mixture of Gal, Man and GlcNAc.


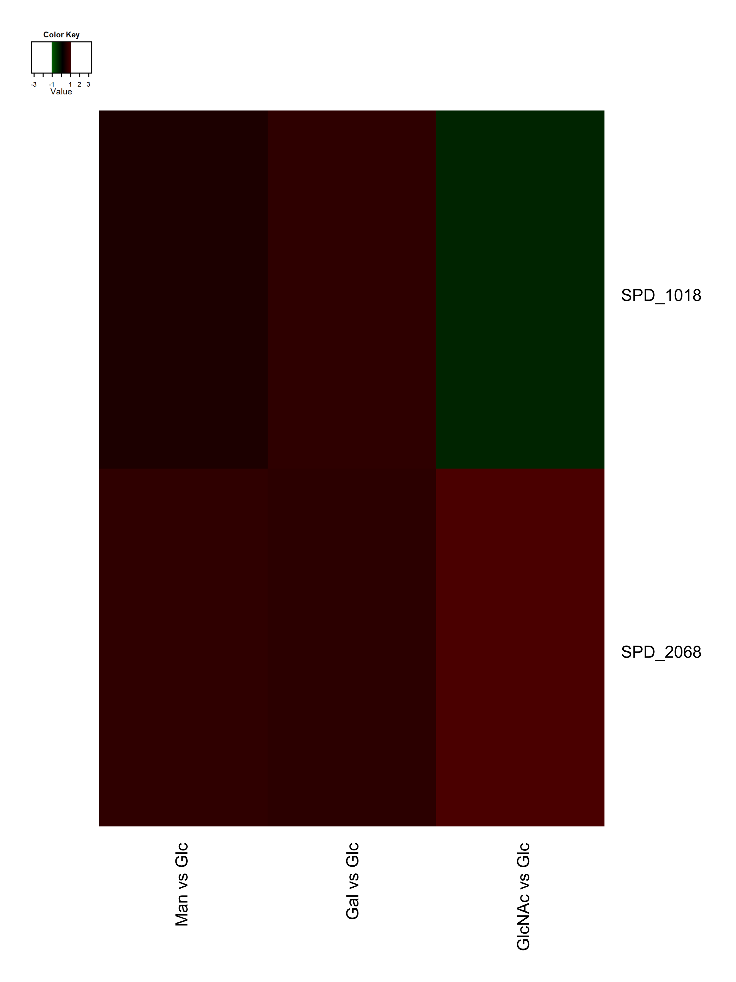


**Figure S3.** **Visual representation of the clustering analysis performed for Gal, Man or GlcNAc *versus* Glc, targeting the selected categories of genes.** Colour scale: ln-ratio of expression. Red, upregulated gene in the transcriptome analysis; green, downregulated gene in the transcriptome analysis. The figure was generated targeting the gene categories of interest (glycolysis, pyruvate metabolism, sugar dedicated transporters and catabolic genes as reviewed by Paixão *et al.*, (2015) and classical virulence factors). According to NCBI annotation: SPD_1018, immunoglobulin A1 protease (*iga*); SPD_2068, serine protease. Glc, glucose; Man, mannose; Gal, galactose; GlcNAc, N-acetylglucosamine.

## Supplementary Tables

**Table S1. Virulence factors of *Streptococcus pneumoniae*.**

**Table S2. Significantly differentially expressed genes (up- or downregulated) of cells of *S. pneumoniae* D39 grown in CDM supplemented with N-acetylglucosamine as compared to glucose grown cells, determined by DNA microarrays.**

**Table S3. Significantly differentially expressed genes (up- or downregulated) of cells of *S. pneumoniae* D39 grown in CDM supplemented with mannose as compared to glucose grown cells, determined by DNA microarrays.**

**Table S4. Significantly differentially expressed genes (up- or downregulated) of cells of *S. pneumoniae* D39 grown in CDM supplemented with galactose as compared to glucose grown cells, determined by DNA microarrays.**

**Table S5. Intracellular intermediates of sugar-specific catabolic pathways of *S. pneumoniae* determined in resting cells (by *in vivo* NMR) or in growing cells (ethanol extracts), metabolizing N-acetylglucosamine (GlcNAc), mannose (Man) or galactose (Gal).**

**Table S6. Summary of the significantly differentially expressed genes (up- or downregulated) of cells of *S. pneumoniae* D39 grown in CDM supplemented with N-acetylglucosamine (GlcNAc), mannose (Man), galactose (Gal) or in a mixture thereof challenged with a glucose (Glc) pulse and compared to unchallenged cells, determined by DNA microarrays.^a^**

**Table S7. Significantly differentially expressed genes (up- or downregulated) of cells of *S. pneumoniae* D39 grown in CDM supplemented with N-acetylglucosamine challenged with a glucose pulse (at mid-exponential phase of growth) as compared to unchallenged cells, determined by DNA microarrays.**

**Table S8. Significantly differentially expressed genes (up- or downregulated) of cells of *S. pneumoniae* D39 grown in CDM supplemented with galactose challenged with a Glc pulse (at mid exponential phase of growth) as compared to unchallenged cells, determined by DNA microarrays.**

**Table S9. Significantly differentially expressed genes (up- or downregulated) of cells of *S. pneumoniae* D39 grown in CDM supplemented with mannose challenged with a Glc pulse (at mid-exponential phase of growth) as compared to unchallenged cells, determined by DNA microarrays.**

**Table S10. Significantly differentially expressed genes (up- or downregulated) of cells of *S. pneumoniae* D39 grown in CDM supplemented with a mixture of galactose, mannose and N-acetylglucosamine challenged with a Glc pulse (at mid-exponential phase of growth) as compared to unchallenged cells, determined by DNA microarrays.**

**Table S11. Overrepresentation of COG categories among the significant genes in each condition.**

**Table S12. Clustering of genes significantly, differentially expressed in the microarrays analysis of *S. pneumoniae* D39 grown in galactose, mannose, N-acetylglucosamine or in a mixture thereof challenged with glucose versus unchallenged cells.**

**Table S13. Clustering of genes significantly, differentially expressed in the microarrays analysis of *S. pneumoniae* D39 grown in galactose, mannose or N-acetylglucosamine versus glucose.**
